# Chokeberry Juice Containing Polyphenols Does Not Affect Cholesterol or Blood Pressure but Modifies the Composition of Plasma Phospholipids Fatty Acids in Individuals at Cardiovascular Risk

**DOI:** 10.3390/nu11040850

**Published:** 2019-04-15

**Authors:** Biljana Pokimica, María-Teresa García-Conesa, Manja Zec, Jasmina Debeljak-Martačić, Slavica Ranković, Nevena Vidović, Gordana Petrović-Oggiano, Aleksandra Konić-Ristić, Maria Glibetić

**Affiliations:** 1 Center of Research Excellence in Nutrition and Metabolism, Institute for Medical Research, University of Belgrade, 11000 Belgrade, Serbia; biljana.pokimica@hotmail.com (B.P.); manjazecimr@gmail.com (M.Z.); minaizdravko@yahoo.com (J.D.-M.); slavica.rankovic.imr@gmail.com (S.R.); nevenakardum@gmail.com (N.V.); g5petrovic@gmail.com (G.P.-O.); sandrakonic@gmail.com (A.K.-R.); mglibetic@gmail.com (M.G.); 2 Research Group on Quality, Safety and Bioactivity of Plant Foods, Campus de Espinardo, Centro de Edafología y Biología Aplicada del Segura-Consejo Superior de Investigaciones Científicas (CEBAS-CSIC), P.O. Box 164, 30100 Murcia, Spain

**Keywords:** *Aronia*, anthocyanins, polyphenols, obesity, hyperlipidemia, blood pressure, SFA, n-6 PUFA, palmitic acid, cardiovascular risk factors

## Abstract

Chokeberry polyphenols have been suggested to reduce cholesterol and blood pressure and thus protect against cardiovascular diseases (CVD), but the evidence in humans is limited and inconsistent. This randomized double-blinded three-parallel groups trial investigated the changes in various anthropometric and clinical biomarkers, and in plasma phospholipids fatty acids (PPFA) in volunteers at cardiovascular risk after a four-week intervention with 100 mL/day of (1) chokeberry juice with a high-dose of polyphenols (1177.11 mg gallic acid equivalents, GAE); (2) chokeberry juice with a low-dose of polyphenols (294.28 mg GAE) and; (3) a nutritionally matched polyphenol-free placebo drink. Our results indicate that the intake of chokeberry juice containing either the low or the high dose of polyphenols cannot be linked with a reduction in total- and low-density lipoprotein (LDL)cholesterol or in systolic (SBP) and diastolic (DBP) blood pressure in comparison with the consumption of the placebo drink. However, we found evidence of moderate changes in the PPFA, i.e., increased saturated fatty acids (SFA), mostly palmitic acid, and reduced n-6 polyunsaturated fatty acids (PUFA), principally linoleic acid (LA) with the intake of chokeberry against the placebo. These effects may be associated with the polyphenols but we could not differentiate a clear dose-response effect. Further research is still needed to elucidate the contribution of the polyphenolic fraction to the potential cardiovascular effects of the chokeberry and to build up the evidence of its potential benefit via the modulation of PPFA composition.

## 1. Introduction

Cardiovascular diseases (CVD) are a leading cause of death in the world, however, up to 80% of premature CVD can be prevented by lifestyle and dietary changes [1]. These changes can help to regulate classic CVD-associated risk factors, such as increased body mass index (BMI) and (or) waist circumference (WC), high blood pressure, and (or) abnormal levels of glucose and lipids in serum [2]. Oxidized low-density lipoprotein (OxLDL) cholesterol as well as the ratios oxLDL/total cholesterol and oxLDL/low-density lipoprotein (LDL)cholesterol are also considered reliable markers of coronary artery disease [3] and of oxidative damage to lipids [4] and constitute additional CVD risk factors of current interest. Particularly striking is the relationship between CVD and the composition of plasma phospholipid fatty acids (PPFA), a marker of cell membrane fatty acid (FA) profile [5]. Individuals with a higher content of n-3 polyunsaturated FA (PUFA) in plasma had a lower risk of acute coronary events [6] and of thrombotic infarction [7]. More specifically, higher levels of arachidonic acid (AA) in plasma phospholipids have been associated with higher hypertension risk in men [8] and elevated levels of saturated stearic and palmitic acids in plasma phospholipids were directly correlated to abnormal cholesterol levels in a multi ethnicity cohort [9].

Recent meta-analyses show consistent indication of the association between the intake of various polyphenol-containing foods and derived products and beneficial changes on a combination of CVD risk factors [10,11,12]. In particular, the intake of berries containing anthocyanins (ANCs) as well as other polyphenols (chlorogenic acid, flavonoids, phenolic acids) has been linked with moderate reductions in total-cholesterol and of systolic and diastolic blood pressure (SBP, DBP). The analyses also suggest that different factors including the metabolic status of the individuals can largely influence the response to the consumption of these compounds [12]. In particular, there is some recent preliminary evidence that individuals ‘at cardiovascular risk’ may be more likely to gain benefits from increased intake of polyphenols than healthy individuals [13]. Overall, better designed human intervention trials are still needed to unequivocally validate the effects on CVD risk factors of polyphenol-containing plant foods and derived products in specific human subpopulations [14,15,16]. It has also been highlighted the importance of establishing the recommended doses of specific polyphenols that should be consumed to derive maximum benefit [17]. However, very few trials have yet been conducted with well-defined and characterized doses of polyphenol-containing specific products or food sources in comparison with appropriate polyphenol-free placebos. 

The present human four-week intervention study was specifically designed to compare the effects of two (high- and low-polyphenol) doses of a commercial chokeberry (*Aronia melanocarpa*) juice vs. a polyphenol-free nutritionally matched placebo drink in a sample population of individuals at risk for cardiovascular disorders. We examined the effects on a number of classic CVD risk factors: BMI, WC, blood pressure (SBP, DBP), and fasting serum levels of glucose (FSG), total-cholesterol, LDL-cholesterol, oxLDL, high-density lipoprotein (HDL) cholesterol, and triglycerides (TAGs). We additionally investigated the composition of PPFA before and after intervention as well as potential changes in the activity of the desaturases delta-5 and delta-6 involved in the endogenous metabolism of FA. The overall aim was to further contribute to the validation in humans of the cardiovascular benefits associated with the intake of polyphenol-containing chokeberry juice in comparison with an appropriate placebo and to determine whether there was a dose-response effect. 

## 2. Materials and Methods

### 2.1. Intervention Drinks

The test drink used in this study was a chokeberry juice (AMJ) kindly donated by Nutrika LTD (Belgrade, Serbia) and registered at the Serbian Ministry of Health as a dietary supplement. A placebo drink (PLB), also elaborated by Nutrika, with a similar appearance (color and flavor) and the same nutritional composition (sugars, minerals, vitamins, organic acids) as AMJ, but without polyphenols [18], was used in this trial as the control. This placebo was also safe for human consumption (according to the EU Directive 94/36/EC on quantities allowed for Liquid Food Supplements) and had been shown to cause no significant changes to several CVD risk factors in a previous pilot human study [18]. An additional intervention drink was produced by dilution of the AMJ with the PLB (ratio 1:3) (AMJ_d_). The final total polyphenol content of the drinks was determined by the Folin-Ciocalteu method and reported to be 1177.11 (AMJ, high-dose) and 294.28 (diluted AMJ_d_, low-dose) mg of GAE/100 mL [19]. The content of total cyanidin-3-glucoside equivalents as determined by the pH differential method [20] was 113.3 mg/100 mL and 28.3 mg/100 mL in the AMJ and AMJd, respectively. During the course of the study, the participants were instructed to keep the beverages in the refrigerator in order to preserve the stability of the polyphenols as previously reported in a similar study with chokeberry juice [21].

### 2.2. Enrollment of the Volunteers and Study Design

This trial was a monocentric, randomized, parallel, placebo-controlled, double-blinded, four-week nutritional intervention in free-living adults. The study protocol adhered to the regulations of the 1975 Declaration of Helsinki, and was approved by the Clinical Hospital Center in Zemun (Belgrade, Serbia), Ethics Committee Approval, No.: 2125, 2013. This study is part of a larger study registered at ClinicalTrials.gov as NCT02800967. Participants were recruited via newspaper advertisement or at the clinic. Interested subjects were invited for a group conversation with the research team where all of the details of the study were explained. After an interview and before the commencement of the study, all eligible volunteers were given the written informed consent to sign. The exclusion criteria were: presence or history of CVD or other chronic disease, type-2 diabetes, cancer, asthma, gastrointestinal disease, taking any medication or dietary supplements, known allergy to berries, smoking, very low blood pressure (< 90/50 mm Hg), pregnancy, lactation, blood donation 16 weeks before the start of the study, or parallel participation in another clinical trial. All the participants were of Serbian nationality, white color and Slavic ethnicity and were instructed to continue with their habitual diet and physical activity, but to refrain from consuming berries and (or) berry products, during the course of the study. Also, they were asked to avoid excessive quantities of other foods rich in polyphenols, such as olive oil, green tea, and nuts.

Subjects were considered ‘at cardiovascular risk’ [22] and included in the trial based on the presence of at least one of the following conditions: (i) elevated BMI (overweight and (or) obese people, BMI ≥ 25.0 kg/m^2^) [23,24], and (or) WC (≥80 cm for women, ≥94 cm for men) [25,26]; (ii) blood pressure values above optimal (SBP/DBP ≥ 120/80 mm Hg) [27]; (iii) high FSG (≥5.5 mmol/L or ≥99 mg/dL) [28]; (iv) altered lipids levels, TAGs (≥1.7 mmol/L or ≥150 mg/dL) [29,30], total-cholesterol (≥5.0 mmo/L or ≥193 mg/dL) [29,31], LDL-cholesterol (> 2.6 mmol/L or > 100 mg/dL) [32] and HDL-cholesterol (men, < 1 mmol/L or < 38 mg/dL; women, < 1.2 mmol/L or < 46 mg/dL) [29,30]. 

**Figure 1:**
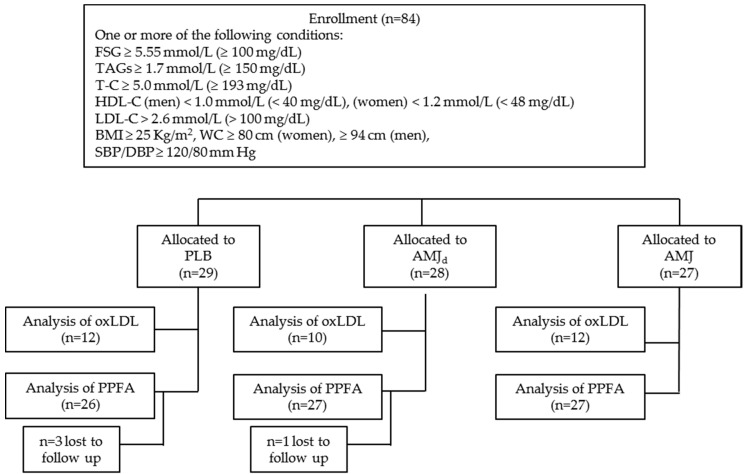
Flow chart indicating the number of participants selected and allocated to the different intervention groups and analyses. FSG: fasting serum glucose; TAGs: triglycerides; T-C: total-cholesterol; HDL-C: High-density lipoprotein-cholesterol; LDL-C: low-density lipoprotein-cholesterol; BMI: body mass index; SBP: systolic blood pressure; DBP: diastolic blood pressure; oxLDL: oxidized low-density lipoprotein; PPFA: plasma phospholipids fatty acids; PLB: placebo; AMJ_d_: polyphenol low-dose chokeberry juice; AMJ: polyphenol high-dose chokeberry juice.

Total-cholesterol levels and SBP were the primary outcomes measured in this study. The sample size was calculated assuming a mean reduction of ~0.3 mmol/L and a SD of ~0.4 mmol/L for cholesterol levels, and a reduction of ~8.0 mm Hg and a SD of ~10 mm Hg for SBP with a power of 80%, a significance level of 0.05, and a 10% drop-out rate [33]. The minimum sample size estimated was *n* = 26–30. A total of 84 subjects were recruited and randomized to receive during four weeks, a daily dose of 100 mL of one of the following three drinks: (1) AMJ (*n* = 27); (2) AMJ_d_ (*n* = 28); (3) PLB (*n* = 29), as depicted in the flow diagram (Figure 1). Compliance was assessed by the empty bottles that the participants returned at the end of the four-week intervention period. 

### 2.3. Data Collection

Habitual dietary intakes throughout the study were assessed using a food frequency questionnaire and two repeated 24-h dietary recalls (24-HDR) performed by a trained dietitian. The volunteers were assisted with a photo-booklet containing reference portions of 125 items, both simple foods and composite dishes to improve the accuracy of the reporting through the estimation of the portion size. The analyses of the data from dietary recalls were performed with the nutritional platform DIET ASSESS & PLAN for standardized food consumption data collection and comprehensive diet evaluation [34]. Total energy (Kcal), main nutrients (fat, protein, carbohydrates), and main types of FA (saturated FA, SFA; monounsaturated FA, MUFA; and PUFA) dietary intakes (g) were calculated using the Serbian Food Composition Database, which is standardized according to the EuroFIR requirements [35]. 

Body weight was determined using a bioelectrical impedance analyzer, TANITA UM072 balance (TANITA Health Equipment Hong Kong, Japan. Ltd.). Participants were asked to remove outer clothing and shoes and instructed to stand in the center of the analyzer’s platform so they could weight evenly on both feet. Height was measured using a height measuring scale. The WC was measured at the umbilicus level, allowing the measurement tape to be horizontal to the floor. Office based SBP and DBP were determined using a digital upper arm electronic blood pressure monitor (OMRON, HEM-907, Omron Healthcare, Tokyo, Japan) in resting subjects in the sitting position. Blood pressure values were obtained in triplicates separated by a 2 min break and the mean values of the three measurements was recorded. All measurements were performed at baseline, and at the end of study. 

### 2.4. Blood Sampling and Biochemical Measurements

Blood samples were taken both at baseline and at the end of the four-week intervention. Blood sampling took place in the morning, between 8 a.m. and 9 a.m., following an overnight fasting (8–12 h after the last meal) and blood was collected in anticoagulant-free tubes and ethylenediaminetetraacetic acid (EDTA)-containing tubes. Serum samples were obtained from anticoagulant-free tubes by centrifugation (2500× *g*, 5 min) and used for the analysis of biochemical variables: FSG, total cholesterol, LDL-cholesterol, HDL-cholesterol and TAGs in a clinical biochemistry analyzer (Cobas c111, Roche, Basel, Switzerland). All analyses were carried out during the same day of extraction. Plasma was separated from erythrocytes in the EDTA tubes by centrifugation (2500× *g*, 5 min) and kept at −80 °C for further analyses. 

### 2.5. Plasma Phospholipid Fatty Acids Composition (PPFA) and Desaturase Activity

Plasma lipids were extracted by the method of Folch et al. [36] using a chloroform-methanol mixture (2:1). Lipids were protected against oxidation by the addition of 2,6-di-tert-butyl-4-methylphenol (10 mg/100 mL) to the mixture of solvents [37]. Phospholipids were then separated from other lipid subclasses on a silica thin-layer chromatography plate using as a solvent system a mixture of petroleum ether, diethyl ether, and glacial acetic acid (87:12:1). Subsequently, the methyl esters of the FA were obtained by direct *trans*-methylation [38] with slight modifications [39]. Briefly, 1.5 mL of hexane and 0.2 mL of NaOH in methanol were added to the phospholipids. The mixture was held at 85 °C for 1 h. The mixture was then cooled down to room T before adding 0.2 mL of 1M H_2_SO_4_ in methanol and maintained at 85 °C for 2 h. Next, the mixture was again cooled down to room T, centrifuged (1860× *g*, 15 min) and dried up using a stream of N_2_. The fatty acid methyl esters were recovered in 10 µL of hexane and injected (1 μL) into a gas-liquid chromatography (Shimadzu chromatograph GC 2014, Tokyo, Japan) equipped with a flame ionization detector and a Rtx 2330 fused silica gel capillary column (60 m × 0.25 mm id × 0.2 μm film thickness) (Restek Co., Bellefonte, PA, USA) [39]. The flow of air was 320 mL/min, the flow of H_2_ was 30 mL/min and the flow of He (carrier gas) was 5 mL/min. The temperature of the flame ionization detector was 260 °C and the temperature of the injection port was 220 °C. The initial temperature of the column was 140 °C and was maintained for 5 min then increased to 220 °C at a rate of 3 °C/min. The final temperature was kept for 20 min. 

Fatty acid methyl esters were identified after 51 min comparing peak retention times with certified calibration mixtures (PUFA-2, Supelco, Bellefonte, PA, USA, and 37 FAMEs mix, Sigma Chemical Co., St. Louis, MO, USA). The main FA detected and quantified were (1) SFA: palmitic acid (16:0) and stearic acid (18:0); (2) MUFA: palmitoleic acid (16:1n-7), oleic acid (18:1n-9), and vaccenic acid (18:1n-7); (3) PUFA: linoleic acid (LA, 18:2n-6), dihomo-γ-linolenic acid (DGLA, 20:3n-6), AA (20:4n-6), adrenic acid (22:4n-6), eicosapentaenoic acid (EPA, 20:5n-3), docosapentaenoic acid (DPA, 22:5n-3), and docosahexaenoic acid (DHA, 22:6n-3). The amounts of individual FA are presented as the relative area percentage of the total (100%) pool of FA detected. 

We used fatty acid product-to-precursor ratios as surrogate parameters for: (i) delta-5 desaturase activity: AA/DGLA (20:4n-6/20:3n-6) and (ii) delta-6 desaturase activity: DGLA/LA (20:3n-6/18:2n-6) [40].

### 2.6. Analysis of oxLDL Levels in Plasma

Serum levels of oxLDL were measured in a subgroup of participants (*n* = 34 subjects, PLB = 12, AMJ = 12, AMJ_d_ = 10; Figure 1) using the enzyme-linked immune-absorbent assay kit 369 (Cell Biolabs Inc., San Diego, CA, USA) and a Thermo Scientific Multiskan FC. Briefly, precipitated LDL was re-suspended in PBS buffer and incubated with the anti-MDA antibody coated plate. After washing, successive steps with biotinylated anti-human ApoB-100 antibody, streptavidin-enzyme conjugate and substrate were carried out following the manufacturer’s instructions. The absorbance was read at 450 nm. The levels of oxLDL were calculated in comparison with an oxLDL standard curve and expressed in ng/mL.

### 2.7. Statistical Analysis

All analyses were performed using SPSS for Windows 22.0 (SPSS Inc., Chicago, IL, USA). The normality of the data for the different groups was tested by the Shapiro-Wilk. Some groups followed a normal distribution, and some did not, thus, for the comparative analysis we chose to apply the more robust non-parametric tests: Kruskal-Wallis (differences between groups) and the Wilcoxon signed rank test (differences intra-groups: post-treatment vs. baseline). When the differences between intervention groups were significant, a post hoc Mann-Whitney U test was performed to determine which groups were significantly different. The main results in the article are reported as medians (IQR). The *p*-values of all the comparisons are indicated. The mean values, SD, 95% CI, and CV (%) are additionally included in Supplementary Tables S1–S6. 

## 3. Results

### 3.1. Baseline Characteristics of the Participants

A total of 84 subjects, 52 women (61.9%) and 32 men (38.1%), mostly young adults and adults (mean age ± SD: 40.6 ± 7.1 years) were enrolled in this trial. Around 82% of the participants had three or more of the clinical conditions applied in the selection, with the majority of them exhibiting elevated levels of LDL-cholesterol (~89%) and (or) extra weight (BMI ≥ 25.0 kg/m^2^) and (or) WC values above the cut-off limits (78% for men and 71% for women). Also, ~58% of the participants had high levels of total-cholesterol and (or) SBP values above the optimum. Overall, these data indicated that the sample population was principally formed by individuals at ‘cardiovascular risk’ with hypercholesterolemia and (or) abdominal obesity, and (or) hypertension. Also, the three groups were homogeneous for the average age although there were a slightly higher proportion of women in the two intervention groups with chokeberry juice.

The baseline characteristics of the participants are all included in Table 1, Table 2, Table 3, Table 4 and Table 5 (also in Supplementary Tables S1–S3). We did not find any significant differences between the three groups for BMI, blood pressure, total and LDL-cholesterol, and FSG although the levels of TAGs were a little higher in the PLB group than in the AMJ_d_ group (Table 1). Most of the individuals, 79%, 81%, and 86% of the PLB, AMJ, and AMJ_d_ groups, respectively, had three or more of the examined conditions. The subpopulation randomly selected for the analysis of oxLDL (*n* = 34) was also constituted mostly by individuals with increased levels of LDL-cholesterol (94.1%), elevated BMI (73.5%), and increased WC (71.4% women and 80.0% men). In this subgroup, above optimal SBP values were also found in 64.7% of the subjects. Baseline oxLDL values as well as the oxidation ratios of cholesterol, i.e., oxLDL/total cholesterol and oxLDL/LDL-cholesterol can be seen in Table 1. None of these values exhibited significant differences between the PLB, AMJ and AMJ_d_ at the beginning of the study. HDL-cholesterol and WC values are presented in men and in women separately, considering the starting cut-off limited different inclusion criteria between the two sexes for these biomarkers (Table 2). We also included here the lipoprotein ratios total-cholesterol/HDL-cholesterol and LDL-cholesterol/HDL-cholesterol. No differences were found between the three groups at the beginning of the study. 

Baseline PPFA composition is included in Table 3 and Table 4. Overall, the PPFA were constituted by a higher proportion of SFA (~47%) and PUFA (~42%) than of MUFA (~11%). Within the PUFA, the proportion of n-6 was ~10-fold higher than that of n-3 (~38% against ~3.8%). The most abundant FA were palmitic acid (~30%) > LA (~24%) > stearic acid (~17%) > AA (~11%). The lowest abundant FA were EPA and adrenic acid (~0.3%–0.4%). We did not find significant differences between the three intervention groups at baseline for most of the FA investigated, although the total % of n-6 PUFA and the ratios n-6 PUFA/n-3 PUFA and AA/EPA were all slightly higher in the intervention groups treated with the chokeberry compared to the placebo group. Estimated delta-5 desaturase activity was approximately 30-fold higher than delta-6 desaturase activity (Table 5). No significant differences between groups were found at baseline.

Regarding habitual dietary intakes of the participants during the study, we did not detect significant differences between the three intervention groups for the intake of total energy, main nutrients, or main types of FA (Table S7). The overall compliance of the participants was ≥98%. 

### 3.2. Responses to Intervention with the Chokeberry Juices and the Placebo Drink: Changes in the Main Anthropometric and Clinical Biomarkers Investigated

Results of the responses to intervention with the chokeberry juices, AMJ_d_ and AMJ, and the PLB for the anthropometric and clinical biomarkers examined in this study are presented in Table 1 (BMI, blood pressure, total cholesterol and LDL-cholesterol, TAGs, FSG, oxLDL) and Table 2 (WC and HDL-cholesterol). All the values are reported as median (IQR) and the *p*-values of all the intragroup and between-group comparisons are indicated. None of the examined biomarkers were seemingly affected by the intervention with the chokeberry juice containing polyphenols. Overall, the changes detected were small (mostly ≤ 5%–10% of baseline values) and we were not able to unequivocally attribute any of those changes to the intake of polyphenol-containing chokeberry juice with sufficient statistical evidence in comparison with the PLB drink. For example, we observed a similar reduction in SBP and DBP following the four-week intake of the chokeberry juices but also with the intake of the PLB drink. On average, the size of this reduction was ~4–5 mm Hg (~4–5% of baseline values) in the three intervention groups. We also observed that the levels of oxLDL as well as those of the ratios oxLDL/T-C and oxLDL/LDL-C were all decreased in a slightly greater proportion after intervention with the chokeberry juices than with the PLB drink but this reduction was significant only in the group treated with the highest dose of polyphenols (AMJ) when compared to baseline values. There were no significant differences between the three groups at the end of the intervention.

### 3.3. Responses to Intervention with the Chokeberry Juices and the Placebo Drink: Changes in PPFA Composition

Comparative analyses of the effects of the intervention with the chokeberry juices, AMJ and AMJ_d_, and with the PLB drink on the composition of SFA and MUFA in PPFA are included in Table 3. The most noticeable change that could be attributed to the chokeberry juice intake was a small but significant increase in the proportion of palmitic acid in the groups that consumed the low-dose and the high-dose polyphenol-containing chokeberry juice, (3.30% and 5.58% increase, respectively) (Table 3). These changes were also almost significantly different (*p* = 0.05) from the increase detected in the PLB group (0.44%) but we were not able to detect any significant difference between the two doses. In parallel to these results, we also observed a sizable (~7%) increase in the proportion of total SFA in the chokeberry-treated groups, although these values did not reach statistical significance in comparison with the PLB group. Stearic acid was similarly increased in the three intervention groups. There were no substantial changes in the proportion of MUFA that could be attributed to the chokeberry juices. 

Changes in the proportions of PUFA in PPFA following intervention with the three drinks are included in Table 4. At the end of the intervention, the consumers of the chokeberry juice had lower proportions of total PUFA, i.e., 7.44% reduction following the intake of the AMJ_d_ and 6.00% reduction after consuming the AMJ, in comparison with those that consumed the PLB drink (1.14% reduction). These changes were paralleled by a reduction of total n-6 PUFA also in these two groups and, more specifically, by the reduction of LA, DGLA, and AA (although only LA showed statistically significant differences between groups). The changes detected in AA were additionally and significantly reflected in a reduction of the ratio AA/EPA only within the groups treated with the chokeberry juice. We were not able to detect substantial differences in the proportion of total n-3 PUFA between the three intervention groups. With regards to the estimated desaturase activities, we did not discern any substantial or significant response to any of the three interventions (Table 5). 

## 4. Discussion

Accumulated evidence of the modulatory effects of polyphenol-containing berries and berry-products on cardiovascular health suggests that these products may reduce total-cholesterol and blood pressure [12]. Despite the large number of studies conducted, publication bias and variability of the results poses some concern about the reliability of these effects as well as the need to continue the research in this area with specific recommendations, i.e., improved study design, detailed specific doses of the polyphenols present in the test products, use of appropriate placebos, improved description of the participants (sex, metabolic status), report less favorable or negative results, and boost the study of the influence of different factors on the effects of these products [13,14]. To further support and try to confirm some of the potential benefits attributed to polyphenol-containing berries, in the present study we have addressed some of those requirements and investigated the effects of a chokeberry juice with two (high- and low-) doses of polyphenols against a nutritionally matched polyphenol-free placebo in a population of individuals characterized by having some cardiovascular risk. All our results, both significant and not-significant, have been included in Table 1, Table 2, Table 3, Table 4 and Table 5 and in Supplementary Tables S1–S6. 

A number of intervention studies have previously investigated the cardiovascular effects of daily doses of polyphenol-containing chokeberry products (juices or extracts) in different subpopulations and have reported a variety of opposed effects in body weight, BMI, WC, blood pressure, and (or) circulating lipids and glucose [21,40,41,42,43,44,45,46]. Overall the most reiterated effects were the reduction of total-cholesterol, LDL-cholesterol, and blood pressure supporting some cardiovascular benefits of the chokeberry, principally, in individuals with some alteration of their metabolic health. The sample population selected for our study was constituted by people with hypercholesterolemia, abdominal obesity, and (or) hypertension and thus can be considered at cardiovascular risk. We were not able to confirm the reduction of total-cholesterol or LDL-cholesterol since we did not find any substantial or significant change in these variables in any of the intervention groups. Regarding blood pressure, previous intervention studies have attributed to the intake of chokeberry, reductions of SBP of up to 11–14 mm Hg and of DBP of 5–8 mm Hg [41,42,43,45], but many of these studies have been carried out without including appropriate placebos. Other studies have reported a more modest decrease in blood pressure (1–3 mm Hg) [44] or even no significant differences [46] in comparison with a placebo control group. More in consonance with these results, we found that SBP and DBP were reduced by only 4–5 mm Hg with the intake of the chokeberry juices but also and very importantly, with the intake of the polyphenol-free nutritionally matched placebo [18]. These results do not support an effect of the chokeberry containing polyphenols on blood pressure and, although we cannot discard that other constituents commonly present in the chokeberry juice and in the placebo, i.e., minerals like potassium [47], may contribute to the lowering of blood pressure, it is conceivable that the observed reduction in blood pressure in the three intervention groups may be the consequence of a ‘placebo’ effect or a time-point difference. 

Ox-LDL and oxidation ratios of cholesterol (oxLDL/LDL-cholesterol and oxLDL/total-cholesterol) are also considered risk factors for atherosclerosis as well as good biomarkers of artery disease with oxLDL and ratios normally being higher in patients than in healthy subjects [3,48]. Increased oxLDL levels are additionally associated with the development of metabolic syndrome [49], diabetes, and hypertension [50,51]. These biomarkers are responsive to therapy with statins by decreasing significantly with independence of the reduction of LDL- and total-cholesterol [3]. In our study, even though we did not detect changes in the total- or LDL-cholesterol, we were able to discern some decrease in the levels of oxLDL (as well as in the oxidation ratios) in the groups treated with the chokeberry juices. This reduction reached significance only in the group exposed to the highest polyphenols dose (post- vs. pre-intervention) and the size of the effects ranged between 17% and 19%. Although these values were not significantly different from those of the placebo group, the reducing effect after consuming the placebo drink was only 4%–7%. We have found in the literature opposed results of the chokeberry against oxLDL, with a recent study finding this biomarker was not altered in mild hyperlipidemic subjects after the intake of a chokeberry extract for 6- and 12-weeks [46], whereas it was reduced by ~25% in patients of myocardial infarction under statin treatment also following consumption of a chokeberry extract [52]. Although the study results of the potential antioxidant effects of polyphenol-containing chokeberry on LDL are still insufficient to claim a true effect, altogether these preliminary results suggest that oxLDL may constitute a target for these compounds that merits further investigation. 

The association between PPFA status and the risk for cardiovascular disorders remains contentious but recent meta-analyses and large cohort studies report the link between specific FA composition and the incidence of obesity [53], type-2 diabetes [54,55], or coronary heart disease [56]. More specifically, palmitic acid, both in serum and in phospholipids, has been associated with increased risk of diabetes [57] and of atrial fibrillation [58]. Also, LA has been negatively associated with type-2 diabetes [55] and metabolic syndrome [59], whereas increased n-3 PUFA (EPA, DHA) as well as total lower n-6 PUFA and LA have been associated with less arterial stiffness [60]. Furthermore, the reduction of AA/EPA may be beneficial in patients with congenital heart disease [61] and the reduction of AA could also be advantageous against inflammatory-based chronic disorders [62]. It has been proposed in dietary guidelines for CVD prevention that reducing SFA intake (to ≤10% of total energy) and replacement by PUFA can lead to important reductions of cardiovascular events (up to 27%) [63]. It has also been reported that dietary changes that promote small reduction (2%–3%) of the intake of SFA and similar small increases in the intake of MUFA for only 12 weeks can promote significant changes in the relative composition of PPFA. The magnitude of these changes was rather small (≤1%) [64]. It thus appears that changes in diet that are accepted to prevent cardiovascular events can be associated with small % of changes in PPFA composition. In this context, and although a link between PPFA and CVD has not yet been established as a clear-cut causative relationship, the observed increase in the relative proportion of SFA in PPFA, especially of palmitic acid, after the intake of the chokeberry juices may be seen as deleterious for CVD. In contrast, the observed reduction of total n-6 PUFA and of LA may be considered beneficial against the stiffness of the arteries [60] and a reduction of AA and of AA/EPA may also be beneficial. Previous studies conducted with polyphenol containing juices have already indicated the occurrence of changes in the PPFA composition and thus, the daily consumption of a pomegranate juice rich in ANCs (among other polyphenols) for six weeks also increased the proportion of SFA and reduced the n-6 PUFA (AA) in the PPFA in women with metabolic syndrome [65], supporting our results. Nevertheless, other published reports give evidence of variability in the effects and, for example, the same chokeberry juices used in our study caused different changes in the PPFA composition in a rat model [19] and a similar chokeberry juice to the one used in our study (containing ~600 mg/100 mL of polyphenols) had an overall weak impact on the serum phospholipid composition of young sportive subjects [40]. Additionally, in humans, a polyphenol-rich chokeberry juice [21] and a glucomannan-enriched chokeberry drink [42] significantly altered the FA composition of the erythrocytes membranes. In both cases, an increase of n-3 PUFA and DHA and a reduction of MUFA were reported. All these studies show preliminary evidence of changes that may be occurring in the FA profile in different body compartments following intervention for several weeks with berry juices containing polyphenols. However, these effects are still scarce and inconsistent, they do not seem to be product-specific, and cannot yet be unequivocally attributable to the polyphenols. Further, the variability in the results may be influenced by the specific phospholipids compartment investigated, the health status, or the organism investigated. Whether any of these effects are maintained in the long term and may have a true impact in the development and (or) prevention of CVD remains to be determined, however, overall, these results are indicative of changes in the PPFA with the intake of chokeberry containing polyphenols worth investigating more extensively. 

Regarding potential mechanisms of action, changes in the PPFA composition have been recognized to reflect partially the dietary intake of FA but also their endogenous metabolism, which is regulated by enzymes such as elongases and delta-5 and delta-6 desaturases [66]. In agreement with previous results [40], we did not find any significant effect of the chokeberry juices on any of these two desaturases and thus, we cannot attribute the changes observed in the n-6 PPFA to a direct effect on the endogenous synthesis of DGLA or AA via a modulatory effect on these enzymes. It is plausible that changes in the habitual diet may have accounted for some of the reported FA changes. We recorded the diet of the participants throughout the study and estimated that there were no significant differences regarding dietary intakes of total SFA, MUFA, and PUFA between the three intervention groups (Table S7) and thus, we were not able to estimate to what extent our results were influenced by dietary FA. Nevertheless, we cannot fully exclude the possibility that the intake of specific foods and FA may have an effect on some of the PPFA levels in our study. One additional hypothesis is that polyphenols may alter the absorption/elimination of FA from the diet. The bioavailability of LA, a FA that cannot be synthesized by mammals [67] and precursor for DGLA and AA [66], has been proposed to be reduced by rye polyphenols by decreasing fat absorption and increasing fecal lipid excretion [68]. In our study, LA was reduced after the intake of the chokeberry juices and thus, it is conceivable that the chokeberry juice (or the polyphenols present in it) may have influenced the absorption of LA. 

It has been stated that the doses of polyphenols needed to achieve a significant effect observable over a period of time is an issue that needs to be critically evaluated [17]. Our study was specifically designed for this purpose but despite our efforts to compare between chokeberry with high- and low-polyphenol content, we were not able to discern a clear dose-response effect. A potential explanation may be that the smallest dose used in this study (~300 mg of polyphenols/day) was already sufficient to attain the maximum observable effect. In support of this, it has been estimated that the relationship between a daily exposure to a dose of polyphenols and the reduction of the risk of some CVD such as type-2 diabetes follows a non-linear correlation [69]. Also, the size of the effects is small and differences between the effects attributed to different doses are also small. Nevertheless, some of the effects on PPFA were seen with the chokeberry juices but not with the placebo drink, supporting a role for the polyphenols. Although we did not carry out a full bioavailability study within this trial, some urine samples obtained from several of the volunteers that consumed the highest dose of chokeberry juice (AMJ) were analyzed in a parallel study. The results of this analysis demonstrated the presence of cyanidin in several samples as well as a significant increment of various phenolic metabolites derived from the microbial metabolism of polyphenols [70], i.e., protocatechuic acid-glucuronide, 3,4-dihydroxy-phenylacetic acid, hippuric acid, and some γ-valerolactones and γ-valerolactone glucuronides [71], supporting the absorption and metabolism of the chokeberry juice derived polyphenols and thus, the plausibility of their involvement in the cardiometabolic effects reported here. 

Overall, the results found in our study, as well as in similar previously published ones [21,40,42,43,44,45,46,52,65], show the difficulty in attaining consistent responses of the cardiovascular biomarkers to intervention with dietary polyphenols such as those present in berries and berry derived products. One of the main limitations remains to be the gathering of a sufficient number of individuals per sample population so that the results may reach adequate evidence. This is especially relevant in studies looking at the effects of dietary polyphenols given the small effect sizes observed and the high variability in the responses. Understanding this variability remains essential to fully comprehending their potential benefits against the development of CVD in humans and to identify specific populations that may benefit best from the intake of these products. We still need to elucidate which and how different factors such as sex, BMI, health status, and genetic characteristics influence this variability [14]. Additionally, regarding the duration of the intervention, most published studies looking at the cardiovascular effects of these products have been carried out during a period of several weeks [18,40,42,52,65] and it may be that this is not long enough to cause more substantial and relevant changes. Thus, future studies should be designed with longer intervention periods. 

## 5. Conclusions

Our study provides additional evidence that the daily intake for several weeks of chokeberry juice with either a low- or a high-dose of polyphenols does not have a substantial impact on a range of classic anthropometric and cardiovascular biomarkers in individuals at cardiovascular risk. Our results do not support a reducing effect on total- and LDL-cholesterol but provide evidence of the relevance of using a nutritionally-matched drink without polyphenols to detect potential ‘placebo’ effects, such as the reducing effect on blood pressure.

In addition, our data indicate that the intake of the two doses of polyphenol-containing chokeberry juice is associated with some modifications in the composition of PPFA, principally, increased PA and reduced n-6 PUFA. Even though we were not able to discern a clear dose-response, these results points towards PPFA as a potential responsive target to the consumption of chokeberry juice with polyphenols. We still need to elucidate the specific contribution and the optimal dose of the polyphenols needed. The effects of these compounds on the absorption, distribution, and metabolism of FA also warrant further research.

## Figures and Tables

**Table 1:** Changes in some of the anthropometric and biochemical variables examined in this study following daily intervention for four weeks with the placebo (PLB) drink, the polyphenol low-dose chokeberry juice (AMJ_d_), and the polyphenol high-dose chokeberry juice (AMJ).

	PLB (*n* = 29)	AMJd (*n* = 28)	AMJ (*n* = 27)	*p*-Value (between Groups)
BMI (kg/m^2^)				
Baseline	27.29 (5.64)	26.59 (7.13)	27.38 (5.73)	0.82
End of trial	27.33 (5.46)	26.75 (6.67)	27.00 (5.64)	0.86
Δ (Δ%)	−0.03 (−0.09%)	0.02 (0.26%)	−0.11 (−0.52%)	0.38
*p*-value (intragroup)	0.40	0.58	0.06	
SBP (mm Hg)				
Baseline	120.00 (23.25)	121.00 (14.50)	124.50 (16.00)	0.26
End of trial	113.75 (24.63)	118.75 (16.00)	115.00 (13.00)	0.96
Δ (Δ%)	−4.00 (−3.31%)	−5.25 (−4.58%)	−6.50 (−5.42%)	0.33
*p*-value (intragroup)	0.001	0.06	0.0001	
DBP (mm Hg)				
Baseline	75.00 (19.38)	75.50 (13.00)	77.00 (17.00)	0.36
End of trial	73.00 (23.38)	70.50 (14.50)	72.00 (14.00)	0.89
Δ (Δ%)	−3.25 (−4.57%)	−4.00 (−5.20%)	−4.00 (−4.83%)	0.69
*p*-value (intragroup)	0.006	0.05	0.002	
T-C (mmol/L)				
Baseline	5.37 (1.35)	4.98 (0.95)	5.18 (1.77)	0.44
End of trial	5.38 (1.27)	5.02 (1.36)	5.26 (1.98)	0.46
Δ (Δ%)	−0.04 (−1.52%)	−0.07 (−1.20%)	−0.06 (−1.16%)	0.96
*p*-value (intragroup)	0.39	0.35	0.74	
LDL-C (mmol/L)				
Baseline	3.79 (1.09)	3.29 (1.00)	3.43 (1.72)	0.26
End of trial	3.81 (1.02)	3.27 (1.15)	3.56 (1.59)	0.32
Δ (Δ%)	−0.02 (−1.75%)	0.01 (0.42%)	−0.01 (−0.22%)	0.78
*p*-value (intragroup)	0.49	0.93	0.86	
TAGs (mmol/L)				
Baseline	1.35 (0.62)	0.95 (0.54) ^1^	1.10 (1.10)	0.05
End of trial	1.28 (1.17)	1.13 (0.61)	1.00 (0.75)	0.10
Δ (Δ%)	0.02 (−4.47%)	−0.01 (0.78%)	0.03 (3.33%)	0.97
*p*-value (intragroup)	0.20	0.88	0.78	
FSG (mmol/L)				
Baseline	5.06 (0.57)	4.75 (0.69)	4.86 (0.67)	0.08
End of trial	5.04 (0.84)	4.77 (0.67)	5.10 (0.73)	0.11
Δ (Δ%)	−0.09 (−1.72%)	0.04 (1.09%)	0.18 (3.93%)	0.25
*p*-value (intragroup)	0.37	0.45	0.08	
oxLDL (ng/mL) ^2^				
Baseline	117.00 (46.50)	133.00 (43.75)	129.00 (36.50)	0.46
End of trial	115.00 (50.50)	121.50 (46.00)	110.50 (28.00)	0.58
Δ (Δ%)	−8.00 (−6.85%)	−21.50 (−15.72%)	−21.50 (−16.81%)	0.73
*p*-value (intragroup)	0.53	0.65	0.02	
oxLDL/T-C (ng/mg)				
Baseline	53.16 (21.05)	65.84 (21.13)	65.70 (24.42)	0.22
End of trial	53.62 (20.95)	58.42 (56.73)	45.79 (21.58)	0.52
Δ (Δ%)	−3.44 (−5.59%)	−10.11 (−13.78%)	−9.49 (−18.95%)	0.53
*p*-value (intragroup)	0.58	0.96	0.02	
oxLDL/LDL-C (ng/mg)				
Baseline	76.84 (37.98)	93.94 (45.28)	93.09 (41.28)	0.23
End of trial	74.56 (34.50)	86.73 (109.43)	62.71 (36.46)	0.48
Δ (Δ%)	−3.64 (−4.00%)	−13.66 (−11.45%)	−15.55 (−19.02%)	0.31
*p*-value (intragroup)	0.69	0.80	0.01	

Results are presented as the median (IQR); Δ: median of (end-baseline) values; Δ%: median of ((end-baseline values)/baseline) × 100. Between groups differences were estimated by the non-parametric test of Kruskal-Wallis and intragroup differences by the Wilcoxon signed rank test. A Mann-Whitney test was applied when there were differences between groups. ^1^: different from placebo by Mann-Whitney test; ^2^: *n* = 12 (PLB), *n* = 10 (AMJ_d_), *n* = 12 (AMJ). BMI: body mass index; SBP: systolic blood pressure; DBP: diastolic blood pressure; T-C; total cholesterol; LDL-C: low-density lipoprotein (LDL) cholesterol; TAGs: triglycerides; FSG: fasting serum glucose; oxLDL: oxidized LDL.

**Table 2:** Changes in waist circumference (WC) and HDL-cholesterol in women and men following daily intervention for four weeks with the placebo (PLB) drink, the polyphenol low-dose chokeberry juice (AMJd), and the polyphenol high-dose chokeberry juice (AMJ).

	PLB (*n* = 14)	AMJd (*n* = 21)	AMJ (*n* = 17)	*p*-Value (between Groups)
Women				
WC (cm)				
Baseline	87.00 (19.00)	83.00 (15.50)	85.00 (27.50)	0.86
End of trial	86.00 (24.25)	83.00 (15.50)	88.00 (24.00)	0.72
Δ (Δ%)	−1.50 (−1.75%)	−1.00 (−1.25%)	−1.00 (−1.10%)	0.78
*p*-value (intragroup)	0.09	0.07	0.28	
HDL-C (mmol/L)				
Baseline	1.87 (0.52)	1.74 (0.64)	1.79 (0.63)	0.52
End of trial	1.72 (0.52)	1.63 (0.61)	1.66 (0.67)	0.57
Δ (Δ%)	−0.09 (−4.48%)	−0.16 (−8.74%)	−0.03 (−1.41%)	0.18
*p*-value (intragroup)	0.21	0.02	0.42	
T-C/HDL-C				
Baseline	3.13 (1.35)	2.96 (0.78)	3.02 (1.70)	0.77
End of trial	3.21 (1.03)	3.21 (1.51)	3.12 (1.55)	0.86
Δ (Δ%)	0.08 (2.74%)	0.14 (7.10%)	−0.03(−1.53%)	0.26
*p*-value (intragroup)	0.64	0.11	0.59	
LDL-C/HDL-C				
Baseline	2.19 (1.02)	1.94 (0.83)	1.85 (1.48)	0.83
End of trial	2.17 (0.96)	1.90 (1.13)	2.03 (1.14)	0.79
Δ (Δ%)	0.13 (6.39%)	0.11 (6.50%)	−0.03 (−1.25%)	0.48
*p*-value (intragroup)	0.55	0.16	0.87	
	**PLB (*n* = 15)**	**AMJd (*n* = 7)**	**AMJ (*n* = 10)**	***p*-Value (between Groups)**
Men				
WC (cm)				
Baseline	101.00 (11.00)	109.00 (9.00)	100.00 (16.00)	0.36
End of trial	100.50 (15.71)	105.00 (18.00)	99.00 (15.25)	0.45
Δ (Δ%)	−1.00 (−0.99%)	−1.00 (−0.98%)	−2.00 (−1.91%)	0.88
*p*-value (intragroup)	0.04	0.38	0.08	
HDL-C (mmol/L)				
Baseline	1.25 (0.27)	1.60 (0.71)	1.47 (0.46)	0.21
End of trial	1.18 (0.30)	1.45 (0.46)	1.47 (0.70)	0.31
Δ (Δ%)	−0.07 (−4.82%)	−0.10 (−6.25%)	−0.04 (−2.45%)	0.68
*p*-value (intragroup)	0.03	0.11	0.44	
T-C/HDL-C				
Baseline	4.01 (2.21)	3.26 (0.99)	4.04 (1.95)	0.40
End of trial	4.40 (1.79)	3.89 (1.02)	4.24 (2.36)	0.31
Δ (Δ%)	0.43 (9.69%)	0.33 (9.40%)	−0.04 (−1.76%)	0.80
*p*-value (intragroup)	0.06	0.31	0.72	
LDL-C/HDL-C				
Baseline	2.84 (1.74)	2.34 (0.52)	2.86 (1.36)	0.31
End of trial	3.11 (1.35)	2.54 (1.01)	3.10 (2.12)	0.38
Δ (Δ%)	0.25 (8.77%)	0.27 (13.19%)	0.23 (8.90%)	0.96
*p*-value (intragroup)	0.27	0.31	0.33	

Results are presented as the median (IQR); Δ: median of (end-baseline) values; Δ%: median of ((end-baseline values)/baseline) × 100. WC: waist circumference; T-C; total cholesterol; HDL-C: high-density lipoprotein (HDL) cholesterol. Between groups differences were estimated by the non-parametric test of Kruskal-Wallis and intragroup differences by the Wilcoxon signed rank test.

**Table 3:** Changes in the levels of saturated (SFA) and monounsaturated (MUFA) fatty acids following daily intervention for four weeks with the placebo (PLB) drink, the polyphenol low-dose chokeberry juice (AMJd) and the polyphenol high-dose chokeberry juice (AMJ).

	PLB (*n* = 26)	AMJd (*n* = 27)	AMJ (*n* = 27)	*p*-Value (between Groups)
Total saturated fatty acids (%)				
Baseline	47.86 (2.84)	45.93 (3.90)	46.25 (3.41)	0.17
End of trial	48.72 (3.79)	49.36 (3.07)	49.28 (1.27)	0.65
Δ (Δ%)	0.85 (1.75%)	3.18 (6.86%)	3.35 (7.21%)	0.13
*p*-value (intragroup)	0.32	0.002	0.005	
Palmitic acid, 16:0 (%)				
Baseline	30.89 (2.19)	30.06 (2.99)	29.21 (4.21)	0.20
End of trial	30.51 (1.33)	31.67 (1.81)	31.73 (1.87)	0.10
Δ (Δ%)	0.13 (0.44%)	0.93 (3.30%) ^1^	1.69 (5.58%) ^1^	0.05
*p*-value (intragroup)	0.77	0.005	0.01	
Stearic acid, 18:0 (%)				
Baseline	16.92 (1.96)	16.24 (1.76)	16.93 (1.77)	0.12
End of trial	17.68 (2.53)	17.86 (1.63)	17.60 (1.55)	0.91
Δ (Δ%)	1.03 (6.27%)	1.72 (10.76%)	0.97 (5.78%)	0.07
*p*-value (intragroup)	0.05	0.005	0.002	
Total monounsaturated fatty acids (%)				
Baseline	10.83 (1.56)	10.95 (2.32)	10.86 (1.21)	0.98
End of trial	10.74 (1.75)	10.65 (2.33)	10.65 (2.04)	0.69
Δ (Δ%)	−0.58 (−5.37%)	−0.13 (−1.43%)	−0.05 (−0.56%)	0.71
*p*-value (intragroup)	0.29	0.85	0.96	
Palmitoleic acid, 16:1n-7 (%)				
Baseline	0.62 (0.33)	0.49 (0.27)	0.53 (0.33)	0.39
End of trial	0.56 (0.27)	0.44 (0.35)	0.49 (0.31)	0.74
Δ (Δ%)	−0.02 (−3.47%)	−0.09 (−18.02%)	0.04 (6.92%)	0.70
*p*-value (intragroup)	0.14	0.12	0.56	
Oleic acid, 18:1n-9 (%)				
Baseline	7.71 (1.39)	8.13 (2.02)	7.76 (1.00)	0.96
End of trial	6.95 (1.58)	7.46 (1.61)	7.60 (1.57)	0.25
Δ (Δ%)	−0.60 (−7.81%)	−0.46 (−6.07%)	0.16 (2.03%)	0.25
*p*-value (intragroup)	0.02	0.08	0.65	
Vaccenic acid, 18:1n-7 (%)				
Baseline	2.44 (0.62)	2.23 (0.89)	2.63 (0.63)	0.47
End of trial	2.84 (0.86)	2.59 (0.89)	2.67 (0.80)	0.45
Δ (Δ%)	0.54 (19.80%)	0.26 (11.18%)	0.04 (1.49%)	0.29
*p*-value (intragroup)	0.002	0.06	0.18	

Results are presented as the median (IQR); Δ: median of (end-baseline) values; Δ%: median of ((end-baseline values)/baseline) × 100. Between group differences were estimated by the non-parametric test of Kruskal-Wallis and intragroup differences by the Wilcoxon signed rank test. The Mann-Whitney test was applied when there was difference between groups. ^1^: different from placebo (no difference between AMJ and AMJ_d_) by the Mann-Whitney test.

**Table 4:** Changes in the levels of polyunsaturated fatty acids (PUFAs) following daily intervention for four weeks with the placebo (PLB) drink, the polyphenol low-dose chokeberry juice (AMJd), and the polyphenol high-dose chokeberry juice (AMJ).

	PLB (*n* = 26)	AMJd (*n* = 27)	AMJ (*n*= 27)	*p*-Value (between Groups)
Total polyunsaturated fatty acids (%)				
Baseline	41.15 (3.23)	43.46 (2.99)	42.63 (3.35)	0.14
End of trial	40.51 (3.63)	40.14 (2.53)	40.19 (1.81)	0.36
Δ (Δ%)	−0.46 (−1.14%)	−3.15 (−7.44%) ^1^	−2.63 (−6.00%) ^1^	0.01
*p*-value (intragroup)	0.57	0.001	0.001	
Total n-6 polyunsaturated fatty acids (%)				
Baseline	37.80 (4.53)	39.55 (3.14) ^1^	38.92 (2.94) ^1^	0.02
End of trial	36.39 (2.82)	35.97 (3.04)	35.91 (1.79)	0.62
Δ (Δ%)	−0.13 (−0.33%)	−3.97 (−9.10%) ^1^	−2.89 (−7.44) ^1^	0.004
*p*-value (intragroup)	0.68	0.0005	0.0005	
Linoleic acid, 18:2n-6 (%)				
Baseline	23.27 (4.19)	24.88 (3.39)	23.48 (4.97)	0.15
End of trial	23.22 (3.74)	22.95 (2.88)	22.89 (4.17)	0.74
Δ (Δ%)	0.31 (1.61%)	−2.23 (−8.23%) ^1^	−1.42 (−7.15%)	0.03
*p*-value (intragroup)	0.68	0.001	0.06	
Dihomo-γ linolenic acid, 20:3n-6 (%)				
Baseline	2.99 (1.24)	2.84 (1.55)	2.93 (1.67)	0.64
End of trial	2.54 (1.21)	2.56 (1.33)	2.42 (1.30)	0.91
Δ (Δ%)	−0.02 (−0.94%)	−0.17 (−7.51%)	−0.34 (−12.11%)	0.43
*p*-value (intragroup)	0.34	0.22	0.007	
Arachidonic acid, 20:4n-6 (%)				
Baseline	10.74 (2.95)	10.39 (2.64)	11.37 (3.39)	0.74
End of trial	9.70 (4.07)	10.01 (2.59)	10.21 (3.58)	0.91
Δ (Δ%)	−0.38 (−3.59)	−0.71 (−6.62%)	−0.48 (−6.30%)	0.79
*p*-value (intragroup)	0.34	0.06	0.002	
Adrenic acid, 22:4n-6 (%)				
Baseline	0.40 (0.18)	0.43 (0.17)	0.42 (0.17)	0.59
End of trial	0.30 (0.18)	0.36 (0.15)	0.38 (0.20)	0.63
Δ (Δ%)	−0.06 (−17.10%)	−0.11 (−21.80%)	−0.09 (−21.41%)	0.74
*p*-value (intragroup)	0.07	0.05	0.004	
Total n-3 polyunsaturated fatty acids (%)				
Baseline	4.03 (1.54)	3.58 (1.66)	3.36 (1.52)	0.08
End of trial	3.96 (1.77)	3.47 (1.84)	3.46 (1.45)	0.29
Δ (Δ%)	−0.16 (−4.19%)	0.46 (16.58%)	−0.18 (−5.44%)	0.38
*p*-value (intragroup)	0.87	0.25	0.94	
Eicosapentaenoic acid, 20:5n-3 (%)				
Baseline	0.39 (0.22)	0.26 (0.25)	0.28 (0.19)	0.10
End of trial	0.37 (0.35)	0.40 (0.31)	0.46 (0.31)	0.43
Δ (Δ%)	0.06 (23.53%)	0.11 (44.80%)	0.04 (19.74%)	0.79
*p*-value (intragroup)	0.17	0.05	0.07	
Docosapentaenoic acid, 22:5n-3 (%)				
Baseline	0.60 (0.31)	0.53 (0.26)	0.55 (0.19)	0.22
End of trial	0.53 (0.34)	0.46 (0.22)	0.51 (0.22)	0.80
Δ (Δ%)	−0.07 (−15.18%)	−0.01 (−3.61%)	−0.06 (−11.08%)	0.56
*p*-value (intragroup)	0.12	0.55	0.06	
Docosahexaenoic acid, 22:6n-3 (%)				
Baseline	3.07 (1.09)	2.46 (1.21)	2.58 (1.34)	0.11
End of trial	2.90 (1.40)	2.57 (1.49)	2.68 (1.26)	0.50
Δ (Δ%)	−0.09 (−4.62%)	0.27 (13.88%)	−0.01 (−0.33%)	0.37
*p*-value (intragroup)	0.60	0.43	0.61	
Ratio total n6/n3 polyunsaturated fatty acids				
Baseline	9.21 (3.39)	11.24 (5.01) ^1^	11.67 (4.31) ^1^	0.04
End of trial	8.96 (4.57)	10.29 (5.43)	10.58 (3.92)	0.46
Δ (Δ%)	0.48 (5.37%)	−1.47 (−17.58%)	−0.32 (−2.66%)	0.15
*p*-value (intragroup)	0.60	0.06	0.40	
Ratio Arachidonic/Eicosapentaenoic acid				
Baseline	26.97 (16.39)	35.09 (37.20)	36.68 (23.99) ^1^	0.04
End of trial	23.45 (18.01)	24.05 (21.19)	26.44 (20.99)	0.51
Δ (Δ%)	−3.19 (−13.30%)	−14.97 (−40.15%)	−10.44 (−24.27%)	0.47
*p*-value (intragroup)	0.16	0.008	0.03	
Ratio Arachidonic/Docosahexaenoic acid				
Baseline	3.54 (1.08)	4.37 (1.81)	4.16 (1.62)	0.08
End of trial	3.16 (1.19)	3.73 (1.65)	3.98 (2.28)	0.77
Δ (Δ%)	−0.03 (−0.97%)	−0.63 (−12.82%)	−0.23 (−7.94%)	0.32
*p*-value (intragroup)	0.95	0.10	0.34	

Results are presented as the median (IQR); Δ: median of (end-baseline) values; Δ%: median of ((end-baseline values)/baseline) × 100. Between groups differences were estimated by the non-parametric test of Kruskal-Wallis and intragroup differences by the Wilcoxon signed rank test. Mann-Whitney test was applied when there was difference between groups. ^1^: different from placebo (no difference between AMJ and AMJ_d_) by Mann-Whitney test.

**Table 5:** Changes in delta-5 and delta-6 desaturase activity following daily intervention for four weeks with the placebo (PLB) drink, the polyphenol low-dose chokeberry juice (AMJd), and the polyphenol high-dose chokeberry juice (AMJ).

	PLB (*n* = 26)	AMJd (*n* = 27)	AMJ (n =27)	*p*-Value (between Groups)
Delta-5 desaturase activity				
Baseline	3.40 (1.91)	3.68 (2.30)	3.86 (2.10)	0.75
End of trial	4.14 (1.72)	3.85 (1.79)	4.02 (1.63)	0.91
Δ (Δ%)	−0.09 (−2.30%)	0.12 (3.66%)	0.10 (2.53%)	0.73
*p*-value (intragroup)	0.71	0.90	0.37	
Delta-6 desaturase activity				
Baseline	0.13 (0.05)	0.11 (0.07)	0.13 (0.09)	0.35
End of trial	0.12 (0.06)	0.11 (0.07)	0.11 (0.05)	0.95
Δ (Δ%)	0.001 (1.17%)	0.001 (1.00%)	−0.01 (−7.30%)	0.49
*p*-value (intragroup)	0.39	0.98	0.09	

Activity of the enzymes involved in fatty acid metabolism was estimated based on the ratios: arachidonic acid/dihomo-γ-linolenic acid for delta-5 desaturase and dihomo-γ-linolenic acid/linoleic acid for delta-6 desaturase. Results are presented as the median (IQR); Δ: median of (end-baseline) values; Δ%: median of ((end-baseline values)/baseline) × 100. Between groups differences were estimated by the non-parametric test of Kruskal-Wallis and intragroup differences by the Wilcoxon signed rank test. The Mann-Whitney test was applied when there was difference between groups.

## Supplementary Materials

The following are available online at https://www.mdpi.com/2072-6643/11/4/850/s1. Table S1. Baseline characteristics of the sample population as distributed into the placebo (PLB) and chokeberry (AMJ and AMJ_d_) intervention groups. Table S2. Baseline waist circumference and HDL-cholesterol values in women and men as distributed in the three intervention subgroups: placebo (PLB) and chokeberry juices (AMJ and AMJ_d_). Table S3. Baseline plasma phospholipid fatty acid composition and desaturase activity for the three intervention groups: placebo (PLB) and chokeberry juices (AMJ and AMJ_d_). Table S4. Overall changes in the levels of the main biomarkers examined in this study in the placebo (PLB) and chokeberry (AMJ and AMJ_d_) intervention groups. Table S5. Changes in the waist circumference and HDL-cholesterol values of women and men in the three intervention subgroups: placebo (PLB) and chokeberry juices (AMJ and AMJd). Table S6. Changes in the levels of plasma phospholipids fatty acids and desaturase activities in the placebo (PLB) and chokeberry (AMJ and AMJ_d_) intervention groups. Table S7. Habitual daily intake of total energy, main nutrients, and fatty acids of the sample population included in the study and distributed into the placebo (PLB) and chokeberry (AMJ and AMJ_d_) intervention groups.
