# Supplementary material for: Chokeberry Juice Containing Polyphenols Does Not Affect Cholesterol or Blood Pressure but Modifies the Composition of Plasma Phospholipids Fatty Acids in Individuals at Cardiovascular Risk
Source: Nutrients. 2019 Apr 15;11(4):850. doi: 10.3390/nu11040850 (PMC6520894; doi:10.3390/nu11040850)
Supplement: Supplementary file 1 [file nutrients-11-00850-s001.pdf]

Supplementary material

Chokeberry juice containing polyphenols does not affect cholesterol or blood pressure but modifies the composition of plasma phospholipids fatty acids in individuals at cardiovascular risk

Biljana Pokimica[1], María-Teresa García-Conesa[2,*], Manja Zec[1], Jasmina Debeljak-Martačić[1], Slavica Ranković[1], Nevena Vidović[1], Gordana Petrović-Oggiano[1], Aleksandra Konić-Ristić[1], and Maria Glibetić[1]

Table S1. Baseline characteristics of the sample population as distributed into the placebo (PLB) and chokeberry (AMJ and AMJ_d) intervention groups.

	PLB (n=29)	AMJ (n=27)	AMJ_d (n=28)	*p*-value (between groups)
Age				
Mean ± SD	39.00 ± 6.56	42.28 ± 7.45	40.75 ± 7.13	
95% CI	36.61 – 41.39	39.45 – 45.10	38.11 – 43.39	0.25
CV (%)	16.83	17.72	17.49	
Sex (% women)	48.3	62.9	75	-
Body mass index (Kg/m^2)				
Mean ± SD	28.02 ± 5.49	28.46 ± 4.09	28.40 ± 5.20	
95% CI	26.02 – 30.02	26.92 – 30.00	26.47 – 30.32	0.82
CV (%)	19.59	14.35	18.31	
Systolic blood pressure (mm Hg)				
Mean ± SD	123.78 ± 20.37	125.89 ± 11.67	120.23 ± 9.78	
95% CI	116.36 – 131.19	121.49 – 130.29	116.61 – 123.85	0.26
CV (%)	16.45	9.27	8.13	
Diastolic blood pressure (mm Hg)				
Mean ± SD	76.29 ± 13.60	79.09 ± 9.90	74.89 ± 9.68	
95% CI	71.34 – 81.24	75.36 – 82.83	71.31 – 78.48	0.36
CV (%)	17.83	12.52	12.92	
Total cholesterol (mmol/L)				
Mean ± SD	5.41 ± 1.05	5.32 ± 1.09	5.11 ± 0.87	
95% CI	5.03 – 5.79	4.91 – 5.72	4.79 – 5.43	0.44
CV (%)	19.48	20.43	17.04	
LDL cholesterol (mmol/L)				
Mean ± SD	3.77 ± 0.99	3.60 ± 1.08	3.40 ± 0.81	
95% CI	3.41 – 4.13	3.19 – 4.01	3.10 – 3.70	0.26
CV (%)	26.22	30.11	23.66	
Triglycerides (mmol/L)				
Mean ± SD	1.44 ± 0.83	1.27 ± 0.73	1.07 ± 0.57	
95% CI	1.14 – 1.75	0.99 – 1.55	0.85 – 1.28	0.05
CV (%)	57.38	57.61	53.52	
Fasting serum glucose (mmol/L)				
Mean ± SD	5.28 ± 0.80	4.94 ± 0.50	4.87 ± 0.52	
95% CI	4.99 – 5.57	4.75 – 5.13	4.68 – 5.06	0.08
CV (%)	15.18	10.17	10.58	
oxLDL (ng/mL)				
Mean ± SD	114.75 ± 32.66	131.00 ± 24.01	126.30 ± 21.74	
95% CI	96.27 – 133.23	117.41 – 144.59	112.83 – 139.77	0.46
CV (%)	28.46	18.33	17.21	
oxLDL/total cholesterol (ng/mg)				
Mean ± SD	54.13 ± 16.62	62.77 ± 13.12	66.34 ± 14.01	
95% CI	44.73 – 63.54	55.34 – 70.19	57.66 – 75.02	0.22
CV (%)	30.70	20.90	21.11	
oxLDL/LDL cholesterol (ng/mg)				
Mean ± SD	78.51 ± 26.61	91.54 ± 27.11	97.74 ± 27.04	
95% CI	63.45 – 93.56	76.20 – 106.88	80.98 – 114.50	0.23
CV (%)	33.90	29.61	27.67	

Values are mean ± standard deviation (SD), 95% confidence interval (CI) and coefficient of variation (CV). Between groups differences were estimated by the non-parametric test of Kruskal-Wallis. All the *p*-values are indicated. LDL: low-density lipoprotein; oxLDL: oxidized low-density lipoprotein.

Table S2. Baseline waist circumference and HDL-cholesterol values in women and men as distributed in the three intervention subgroups: placebo (PLB) and chokeberry juices (AMJ and AMJ$_d$).

Women	PLB (n=14)	AMJ (n=17)	AMJ$_d$ (n=21)	*p*-value (between groups)
Waist circumference (cm)				
Mean ± SD	89.57 ± 16.63	90.18 ± 13.55	86.38 ± 10.21	
95% CI	80.86–98.28	83.73 – 96.62	82.01 – 90.75	0.86
CV (%)	18.56	15.03	11.82	
HDL cholesterol (mmol/L)				
Mean ± SD	1.86 ± 0.41	1.71 ± 0.54	1.78 ± 0.42	
95% CI	1.64 – 2.07	1.45 –1.96	1.60 – 1.96	0.52
CV (%)	22.10	31.66	23.87	
Total-cholesterol/HDL cholesterol				
Mean ± SD	3.14 ± 0.71	3.19 ± 0.90	3.02 ± 0.78	
95% CI	2.77 – 3.51	2.76 – 3.62	2.68 – 3.35	0.77
CV (%)	22.65	28.21	25.86	
LDL-cholesterol/HDL-cholesterol				
Mean ± SD	2.13 ± 0.64	2.11 ± 0.78	2.01 ± 0.70	
95% CI	1.80 – 2.47	1.74 – 2.47	1.71 – 2.31	0.83
CV (%)	30.08	36.89	34.91	
Men	PLB (n=15)	AMJ (n=10)	AMJ$_d$ (n=7)	
Waist circumference (cm)				
Mean ± SD	100.00 ± 8.47	100.80 ± 12.44	105.43 ± 7.21	
95% CI	95.71 – 104.29	93.45 – 108.15	100.09–110.77	0.36
CV (%)	8.47	12.34	6.84	
HDL cholesterol (mmol/L)				
Mean ± SD	1.26 ± 0.23	1.45 ± 0.43	1.45 ± 0.34	
95% CI	1.14 – 1.37	1.19 – 1.71	1.20 – 1.71	0.21
CV (%)	18.17	29.43	23.70	
Total-cholesterol/HDL cholesterol				
Mean ± SD	4.29 ± 1.25	4.25 ± 1.68	3.60 ± 1.00	
95% CI	3.65 – 4.92	3.21 – 5.29	2.86 – 4.34	0.40
CV (%)	29.23	39.44	27.73	
LDL-cholesterol/HDL-cholesterol				
Mean ± SD	3.11 ± 1.16	3.05 ± 1.45	2.51 ± 0.88	
95% CI	2.52 – 3.70	2.15 – 3.95	1.85 – 3.16	0.31
CV (%)	37.41	47.49	35.21	

Values are mean ± standard deviation (SD), 95% confidence interval (CI) and coefficient of variation (CV). Between groups differences were estimated by the non-parametric test of Kruskal-Wallis. All the *p*-values are indicated. HDL: high-density lipoprotein; LDL: low-density lipoprotein.

Table S3. Baseline plasma phospholipid fatty acid composition and desaturase activity for the three intervention groups: placebo (PLB) and chokeberry juices (AMJ and AMJ$_d$).

	PLB (n=26)	AMJ (n=27)	AMJ$_d$ (n=27)	*p*-value (between groups)
Fatty acid composition				
Palmitic acid, 16:0 (%)				
Mean ± SD	30.88 ± 1.54	30.03 ± 2.56	30.29 ± 2.09	
95% CI	30.29 – 31.48	29.06 – 30.99	29.50 – 31.08	0.20
CV (%)	5.00	8.52	6.90	
Palmitoleic acid, 16:1n-7 (%)				
Mean ± SD	0.64 ± 0.21	0.54 ± 0.18	0.58 ± 0.22	
95% CI	0.56 – 0.72	0.47 – 0.61	0.50 – 0.67	0.39
CV (%)	32.36	33.93	38.29	
Stearic acid, 18:0 (%)				
Mean ± SD	16.75 ± 1.32	17.05 ± 1.44	16.24 ± 1.52	
95% CI	16.24 – 17.26	16.51 – 17.60	15.66 – 16.81	0.12
CV (%)	7.86	8.44	9.38	

Oleic acid, 18:1n-9 (%)				
Mean ± SD	8.03 ± 1.07	7.99 ± 1.51	7.92 ± 1.14	
95% CI	7.62 – 8.45	7.42 – 8.55	7.49 – 8.35	0.96
CV (%)	13.30	18.86	14.35	
Vaccenic acid, 18:1n-7 (%)				
Mean ± SD	2.40 ± 0.42	2.46 ± 0.57	2.37 ± 0.60	
95% CI	2.24 – 2.57	2.24 – 2.67	2.15 – 2.60	0.47
CV (%)	17.38	23.33	25.10	
Linoleic acid, 18:2n-6 (%)				
Mean ± SD	23.17 ± 2.78	23.62 ± 3.38	24.96 ± 3.48	
95% CI	22.10 – 24.24	22.35 – 24.90	23.65 – 26.28	0.15
CV (%)	12.01	14.29	13.96	
Dihomo-γ-linolenic acid, 20:3n-6 (%)				
Mean ± SD	3.00 ± 0.84	3.10 ± 1.07	2.83 ± 0.87	
95% CI	2.67 – 3.32	2.69 – 3.50	2.50 – 3.16	0.64
CV (%)	28.12	34.64	30.65	
Arachidonic acid, 20:4n-6 (%)				
Mean ± SD	10.52 ± 1.99	11.19 ± 2.32	10.81 ± 1.77	
95% CI	9.75 – 11.29	10.32 – 12.07	10.14 – 11.48	0.74
CV (%)	18.95	20.70	16.40	
Eicosapentaenoic acid, 20:5n-3 (%)				
Mean ± SD	0.44 ± 0.26	0.32 ± 0.16	0.35 ± 0.23	
95% CI	0.34 – 0.54	0.26 – 0.38	0.26 – 0.44	0.10
CV (%)	59.36	49.31	65.67	
Adrenic acid, 22:4n-6 (%)				
Mean ± SD	0.41 ± 0.13	0.46 ± 0.17	0.45 ± 0.18	
95% CI	0.36 – 0.46	0.39 – 0.52	0.38 – 0.52	0.59
CV (%)	31.63	36.95	40.81	
Docosapentaenoic acid, 22:5n-3 (%)				
Mean ± SD	0.63 ± 0.23	0.57 ± 0.13	0.51 ± 0.16	
95% CI	0.54 – 0.71	0.52 – 0.62	0.45 – 0.57	0.22
CV (%)	36.91	22.30	31.65	
Docosahexaenoic acid, 22:6n-3 (%)				
Mean ± SD	3.12 ± 0.86	2.76 ± 0.86	2.68 ± 0.92	
95% CI	2.79 – 3.45	2.43 – 3.08	2.33 – 3.03	0.11
CV (%)	27.59	31.32	34.28	
Ratio Arachidonic/Eicosapentaenoic acid				
Mean ± SD	30.35 ± 18.06	41.46 ± 17.38	45.25 ± 29.89	
95% CI	23.41 – 37.29	34.90 – 48.02	33.98 – 56.53	0.04
CV (%)	59.50	41.92	66.04	
Ratio Arachidonic/Docosahexaenoic acid				
Mean ± SD	3.57 ± 0.93	4.40 ± 1.43	4.48 ± 1.61	
95% CI	3.21 – 3.92	3.85 – 4.94	3.87 – 5.09	0.08
CV (%)	25.99	32.64	36.06	
Ratio n-6/n-3 polyunsaturated fatty acids				
Mean ± SD	9.57±2.84	11.29±3.42	11.97±3.61	
95% CI	8.42– 10.71	9.94 – 12.64	10.54 – 13.39	0.04
CV (%)	29.28	30.28	30.16	
Total saturated fatty acids (%)				
Mean ± SD	47.63 ± 2.08	47.08 ± 2.90	46.53 ± 3.01	
95% CI	46.83 – 48.43	45.99 – 48.18	45.39 – 47.66	0.17
CV (%)	4.37	6.17	6.47	
Total monounsaturated fatty acids (%)				
Mean ± SD	11.08 ± 1.16	10.98 ± 1.34	10.87 ± 1.45	
95% CI	10.63 – 11.52	10.47 – 11.49	10.33 – 11.42	0.98
CV (%)	10.51	12.21	13.33	
Total polyunsaturated fatty acids (%)				
Mean ± SD	41.29 ± 2.17	42.02 ± 3.04	42.60 ± 3.11	
95% CI	40.46 – 42.12	40.87 – 43.16	41.43 – 43.77	0.14
CV (%)	5.25	7.23	7.29	
Total n-3 polyunsaturated fatty acids (%)				
Mean ± SD	4.19 ± 1.16	3.65 ± 0.95	3.54 ± 1.06	
95% CI	3.74 – 4.63	3.29 – 4.00	3.14 – 3.94	0.08
CV (%)	27.69	26.16	29.89	
Total n-6 polyunsaturated fatty acids (%)				
Mean ± SD	37.10 ± 2.25	38.37 ± 2.63	39.06 ± 3.10	

95% CI	36.24 – 37.97	37.38 – 39.37	37.89 – 40.23	0.02
CV (%)	6.07	6.87	7.94	
Desaturase activity				
Delta-5 desaturase activity				
Mean ± SD	3.88 ± 1.58	4.05 ± 1.60	4.20 ± 1.53	
95% CI	3.27 – 4.48	3.44 – 4.65	3.62 – 4.78	0.75
CV (%)	40.70	39.51	36.39	
Delta-6 desaturase activity				
Mean ± SD	0.13 ± 0.05	0.14 ± 0.06	0.12 ± 0.05	
95% CI	0.12 – 0.15	0.11 – 0.16	0.10 – 0.14	0.35
CV (%)	34.48	42.51	40.01	

Values are mean ± standard deviation (SD), 95% confidence interval (CI) and coefficient of variation (CV). Between groups differences were estimated by the non-parametric test of Kruskal-Wallis. All the *p*-values are indicated.

Table S4. Overall changes in the levels of the main biomarkers examined in this study in the placebo (PLB) and chokeberry (AMJ and AMJ$_d$) intervention groups.

	PLB (n=29)				AMJ (n=27)				AMJ$_d$ (n=28)				
	Baseline	End of treatment	Δ	IG *p*-value	Baseline	End of treatment	Δ	IG *p*-value	Baseline	End of treatment	Δ	IG *p*-value	BG *p*-value
Body mass index (Kg/m^2)													
Mean ± SD	28.02 ± 5.49	27.94 ± 5.60	-0.08 ± 0.36	0.40	28.46 ± 4.09	28.28 ± 4.15	-0.18 ± 0.44	0.06	28.40 ± 5.20	28.33 ± 5.22	-0.07 ± 0.43	0.58	0.86
95% CI	26.02 – 30.02	25.86 – 30.01	-0.21 – 0.05		26.92 – 30.00	26.71 – 29.85	-0.35 – -0.01		26.47 – 30.32	26.39 – 30.26	-0.23 – 0.09		
CV (%)	19.59	20.06			14.35	14.69			18.31	18.44			
Systolic blood pressure (mm Hg)													
Mean ± SD	123.78 ± 20.37	118.48 ± 18.55	-5.50 ± 7.24	0.001	125.89 ± 11.67	116.98 ± 12.34	-8.91 ± 10.73	0.000	120.23 ± 9.78	116.95 ± 11.73	-3.29 ± 8.86	0.06	0.96
95% CI	116.36 –131.19	111.61 – 125.35	-8.18 – -2.82		121.49 –130.29	112.33 – 121.64	-12.96 – -4.86		116.61–123.85	112.60 –121.29	-6.57 – -0.003		
CV (%)	16.45	15.66			9.27	10.55			8.13	10.03			
Diastolic blood pressure (mm Hg)													
Mean ± SD	76.29± 13.60	73.41 ± 13.36	-3.14 ± 5.77	0.006	79.09 ± 9.90	73.94 ± 10.14	-5.15 ± 8.22	0.002	74.89 ± 9.68	72.75 ± 9.92	-2.14 ± 6.19	0.05	0.89
95% CI	71.34– 81.24	68.46 – 78.36	-5.28 – -1.01		75.36 – 82.83	70.12 – 77.77	-8.25 – -2.05		71.31 – 78.48	69.08 – 76.42	-4.44 – 0.15		
CV (%)	17.83	18.20			12.52	13.72			12.92	13.64			
Total cholesterol (mmol/L)													
Mean ± SD	5.41 ± 1.05	5.37 ± 0.75	-0.07 ± 0.56	0.39	5.32 ± 1.09	5.29 ± 1.10	-0.03 ± 0.51	0.74	5.11 ± 0.87	5.06 ± 1.00	-0.05 ± 0.45	0.35	0.46
95% CI	5.03 – 5.79	5.09 – 5.64	-0.27 – 0.14		4.91 – 5.72	4.87 – 5.70	-0.22 – 0.16		4.79 – 5.43	4.69 – 5.43	-0.22 – 0.12		
CV (%)	19.48	13.97			20.43	20.74			17.04	19.80			
LDL-cholesterol (mmol/L)													
Mean ± SD	3.77 ± 0.99	3.69 ± 0.69	-0.08 ± 0.59	0.49	3.60 ± 1.08	3.61 ± 1.06	0.02 ± 0.51	0.86	3.40 ± 0.81	3.39 ± 1.00	-0.01 ± 0.49	0.93	0.32
95% CI	3.41 – 4.13	3.44 – 3.95	-0.30 – 0.13		3.19 – 4.01	3.22 – 4.01	-0.17 – 0.20		3.10 – 3.70	3.02 – 3.76	-0.19 – 0.17		
CV (%)	26.22	18.77			30.11	29.20			23.66	29.39			
Triglycerides (mmol/L)													
Mean ± SD	1.44 ± 0.83	1.57 ± 1.02	0.12 ± 0.55	0.20	1.27 ± 0.73	1.26 ± 0.79	-0.01 ± 0.58	0.78	1.07 ± 0.57	1.05 ± 0.56	-0.01 ± 0.35	0.88	0.10
95% CI	1.14 – 1.75	1.20 – 1.95	-0.08 –0.33		0.99 – 1.55	0.97 – 1.56	-0.23 – 0.21		0.85 – 1.28	0.85 – 1.26	-0.14 – 0.12		
CV (%)	57.8	64.55			57.61	62.23			53.52	53.39			
Fasting serum glucose (mmol/L)													
Mean ± SD	5.28 ± 0.80	5.29 ± 0.75	-0.01 ± 0.33	0.37	4.94 ± 0.50	5.08 ± 0.55	0.14 ± 0.41	0.08	4.87 ± 0.52	4.97 ± 0.69	0.09 ± 0.42	0.45	0.11
95% CI	4.99 – 5.57	5.01 – 5.57	-0.13 – 0.11		4.75 – 5.13	4.88 – 5.29	-0.02 – 0.29		4.68 – 5.06	4.71 – 5.22	-0.06 – 0.25		
CV (%)	15.18	14.19			10.17	10.73			10.58	13.94			
oxLDL (ng/mL)													
Mean ± SD	114.75 ±32.66	108.58 ± 32.25	-6.17 ± 53.69	0.53	131.00 ± 24.01	110.58 ± 17.75	-20.42 ± 26.45	0.02	126.30 ± 21.74	119.20 ± 30.67	-7.10 ± 40.63	0.65	0.58
95% CI	96.27 – 133.23	90.34 – 126.83	-36.54 –24.21		117.41 –144.59	100.54 – 120.63	-35.38 – -5.45		112.83 – 139.77	100.19 – 138.21	-32.28 –18.08		
CV (%)	28.46	29.70			18.33	16.05			17.21	25.73			
oxLDL/total cholesterol (ng/mg)													
Mean ±SD	54.13 ± 16.62	52.11± 16.88	-2.02 ± 23.66	0.58	62.77 ± 13.12	51.92 ± 14.31	-10.84 ±12.80	0.02	66.34 ± 14.01	66.35 ± 30.55	0.01 ± 27.06	0.96	0.52
95% CI	44.73 – 63.54	42.56 – 61.66	-15.41– 11.37		55.34 –70.19	43.83 – 60.02	-18.09– -3.60		57.66 – 75.02	47.41 – 85.29	-16.76 – 16.78		
Median	53.16	53.62	-3.44		65.70	45.79	-9.49		65.84	58.42	-10.11		
CV (%)	30.70	32.39			20.90	27.56			21.11	46.05			
oxLDL/LDL cholesterol (ng/mg)													

Mean ± SD	78.51 ± 26.61	76.14 ± 25.69	-2.36 ± 34.66		91.54 ± 27.11	73.35 ± 20.96	-18.19±19.98		97.74 ±27.04	107.70 ± 64.23	9.96 ± 55.56		
95% CI	63.45 – 93.56	61.61– 90.68	-21.97 –17.25	0.69	76.20 –106.88	61.49 – 85.21	-29.50 – -6.89	0.01	80.98 –114.50	67.89 –147.51	-24.48 –44.40	0.80	0.48
CV (%)	33.90	33.73			29.61	28.58			27.67	59.63			

Values are mean ± standard deviation (SD), 95% confidence interval (CI) and coefficient of variation (CV). Change (Δ) is post-baseline. Intragroup differences (IG) were estimated by the non-parametric Wilcoxon test and between groups (BG) differences were estimated by the non-parametric Kruskal-Wallis test. All the *p*-values are indicated. LDL: low-density lipoprotein; oxLDL: oxidized low-density lipoprotein.

Table S5. Changes in the waist circumference and HDL-cholesterol values of women and men in the three intervention subgroups: placebo (PLB) and chokeberry juices (AMJ and AMJd).

	PLB				AMJ				AMJ$_d$				
	Baseline	End of treatment	Δ	IG *p*-value	Baseline	End of treatment	Δ	IG *p*-value	Baseline	End of treatment	Δ	IG *p*-value	BG *p*-value
**Women**	PLB (n=14)				AMJ (n=17)				AMJd (n=21)				
Waist circumference (cm)													
Mean ± SD	89.57 ± 16.63	88.86 ± 16.06	-0.71 ± 4.76		90.18 ± 13.55	89.35 ± 13.21	-0.82 ± 2.79		86.38 ± 10.21	85.38 ± 10.05	-1.00 ± 2.32		
95% CI	80.86 – 98.28	80.45 – 97.27	-3.21 – 1.78	0.09	83.73 – 96.62	83.07 – 95.63	-2.15 – 0.50	0.28	82.01 – 90.75	81.08 – 89.68	-1.99 – -0.01	0.07	0.72
CV (%)	18.56	18.07			15.03	14.78			11.82	11.77			
HDL cholesterol (mmol/L)													
Mean ± SD	1.86 ± 0.41	1.77 ± 0.44	-0.09 ± 0.24		1.71 ± 0.54	1.69 ± 0.51	-0.01 ± 0.17		1.78 ± 0.42	1.65 ± 0.45	-0.13 ± 0.22		
95% CI	1.64 – 2.07	1.54 – 2.00	-0.21 – 0.04	0.21	1.45 – 1.96	1.45 – 1.94	-0.09 – 0.07	0.42	1.60 – 1.96	1.46 – 1.85	-0.22 – -0.03	0.02	0.57
CV (%)	22.10	24.75			31.66	30.41			23.87	27.45			
Total cholesterol/HDL													
Mean ± SD	3.14 ± 0.71	3.20 ± 0.70	0.06 ± 0.36		3.19 ± 0.90	3.15 ± 1.01	-0.04 ± 0.38		3.02 ± 0.78	3.21 ± 0.98	0.20 ± 0.53		
95% CI	2.77 – 3.51	2.83 – 3.57	-0.12 – 0.25	0.64	2.76 – 3.62	2.68 – 3.63	-0.22 – 0.14	0.59	2.68 – 3.35	2.79 – 3.63	-0.03 – 0.42	0.11	0.86
CV (%)	22.65	21.87			28.21	31.91			25.86	30.52			
LDL/HDL													
Mean ± SD	2.13 ± 0.64	2.18 ± 0.66	0.05 ± 0.32		2.11 ± 0.78	2.11 ± 0.89	0.001 ± 0.36		2.01 ± 0.70	2.13 ± 0.87	0.12 ± 0.39		
95% CI	1.80–-2.47	1.84–2.53	-0.12–0.22	0.55	1.74 – 2.47	1.68 – 2.53	-0.17 – 0.17	0.87	1.71 – 2.31	1.76 – 2.50	-0.04 – 0.29	0.16	0.79
CV (%)	30.08	30.09			36.89	42.08			34.91	40.81			
**Men**	PLB (n=15)				AMJ (n=10)				AMJ$_d$ (n=7)				
Waist circumference (cm)													
Mean ± SD	100.00 ± 8.47	98.71 ± 9.40	-1.29 ± 1.98		100.80 ± 12.44	99.00 ± 11.66	-1.80 ± 2.86		105.43 ± 7.21	103.86 ± 8.88	-1.57 ± 3.36		
95% CI	95.71 – 104.29	93.79 – 103.64	-2.32 – -0.25	0.04	93.45 – 108.15	92.11 – 105.89	-3.49 – -0.11	0.08	100.09 – 110.77	97.28 – 110.43	-4.06 – 0.92	0.38	0.45
CV (%)	8.47	9.52			12.34	11.78			6.84	8.55			
HDL cholesterol (mmol/L)													
Mean ± SD	1.26 ± 0.23	1.18 ± 0.22	-0.08 ± 0.15	0.03	1.45 ± 0.43	1.39 ± 0.38	-0.07 ± 0.19	0.44	1.45 ± 0.34	1.36 ± 0.30	-0.09 ± 0.14	0.11	0.31
95% CI	1.14 – 1.37	1.06 – 1.30	-0.15 – 0.0005		1.19 – 1.71	1.15 – 1.62	-0.19 – 0.06		1.20 – 1.71	1.13 – 1.58	-0.20 – 0.01		

CV (%)	18.17	19.01			29.43	27.53			23.70	22.43			
Total cholesterol/HDL													
Mean ± SD	4.29 ± 1.25	4.66 ± 1.14	0.33 ± 0.67	0.06	4.25 ± 1.68	4.53 ± 1.68	0.28 ± 1.09	0.72	3.60 ± 1.00	3.87 ± 0.82	0.26 ± 0.68	0.31	0.31
95% CI	3.65 – 4.92	4.06 – 5.25	-0.02 – 0.68		3.21 – 5.29	3.49 – 5.58	-0.40 – 0.96		2.86 – 4.34	3.26 – 4.47	-0.24 – 0.77		
CV (%)	29.23	24.52			39.44	37.04			27.73	21.25			
LDL/HDL													
Mean ± SD	3.11 ± 1.16	3.30 ± 0.88	0.16 ± 0.64	0.27	3.05 ± 1.45	3.30 ± 1.29	0.25 ± 0.81	0.33	2.51 ± 0.88	2.73 ± 0.79	0.22 ± 0.57	0.31	0.38
95% CI	2.52 – 3.70	2.84 – 3.76	-0.17– 0.50		2.15 – 3.95	2.50 – 4.10	-0.25 – 0.75		1.85 – 3.16	2.14 – 3.32	-0.21 – 0.64		
CV (%)	37.41	26.79			47.49	39.10			35.21	29.14			

Values are mean ± standard deviations (SD), 95% confidence intervals (CI) and the coefficient of variation (CV). Change (Δ) is post-baseline. Intragroup (IG) differences were estimated by the non-parametric Wilcoxon test and between groups (BG) differences were estimated by the non-parametric Kruskal-Wallis test. All the *p*-values are indicated. LDL: low-density lipoprotein; HDL: high-density lipoprotein.

Table S6. Changes in the levels of plasma phospholipids fatty acids and desaturase activities in the placebo (PLB) and chokeberry (AMJ and AMJ_d) intervention groups.

	PLB (n=26)				AMJ (n=27)				AMJ_d (n=27)				
	Baseline	End of treatment	Δ	IG *p*-value	Baseline	End of treatment	Δ	IG *p*-value	Baseline	End of treatment	Δ	IG *p*-value	BG *p*-value
Palmitic acid, 16:0 (%)													
Mean ± SD	30.88 ± 1.54	30.75 ± 1.19	-0.14 ±2.18	0.77	30.03 ± 2.56	31.35 ± 1.25	1.32± 2.67	0.01	30.29 ± 2.09	31.36 ± 1.43	1.07 ± 2.15	0.005	0.10
95% CI	30.29 – 31.48	30.29 – 31.20	-0.97 –0.70		29.06–30.99	30.87– 31.82	0.31– 2.33		29.50 –31.08	30.82 – 31.90	0.26 – 1.88		
CV (%)	5.00	3.87			8.52	3.99			6.90	4.57			
Palmitoleic acid, 16:1n-7 (%)													
Mean ± SD	0.64 ± 0.21	0.54 ± 0.16	-0.10 ±0.28	0.14	0.54 ± 0.18	0.52 ± 0.20	-0.02 ± 0.23	0.56	0.58 ± 0.22	0.54 ± 0.24	-0.05 ± 0.29	0.12	0.74
95% CI	0.56 – 0.72	0.48 – 0.60	-0.20 – 0.01		0.47 – 0.61	0.44 – 0.59	-0.11 – 0.06		0.50 – 0.67	0.45 – 0.63	-0.16 – 0.07		
CV (%)	32.36	30.59			33.93	39.31			38.29	43.88			
Stearic acid, 18:0 (%)													
Mean ± SD	16.75 ± 1.32	17.55 ± 1.56	0.80 ± 1.77	0.05	17.05 ± 1.44	17.83 ± 1.43	0.77 ± 1.38	0.002	16.24 ± 1.52	17.60 ± 1.47	1.36 ± 2.14	0.005	0.91
95% CI	16.24 –17.26	16.94 – 18.15	0.12 – 1.47		16.51 – 17.60	17.29 – 18.37	0.25 – 1.30		15.66 – 16.81	17.05 – 18.16	0.56 – 2.17		
CV (%)	7.86	8.91			8.44	8.02			9.38	8.35			
Oleic acid, 18:1n-9 (%)													
Mean ± SD	8.03 ± 1.07	7.30 ± 1.72	-0.74 ±1.82	0.02	7.99 ± 1.51	7.76 ± 1.37	-0.23 ± 1.99	0.65	7.92 ± 1.14	7.76 ± 1.60	-0.16 ± 1.53	0.08	0.25
95% CI	7.62 – 8.45	6.63 – 7.96	-1.44 – 0.04		7.42 – 8.55	7.24 – 8.27	-0.98 – 0.52		7.49 – 8.35	7.15 – 8.37	-0.73 – 0.42		
CV (%)	13.30	23.61			18.86	17.66			14.35	20.68			
Vaccenic acid, 18:1n-7 (%)													
Mean ± SD	2.40 ± 0.42	2.86 ± 0.61	0.46 ± 0.64	0.002	2.46 ± 0.57	2.71 ± 0.52	0.25 ± 0.83	0.18	2.37 ± 0.60	2.75 ± 0.71	0.38 ± 1.02	0.06	0.45
95% CI	2.24 – 2.57	2.63 – 3.10	0.21 – 0.70		2.24 – 2.67	2.51 – 2.91	-0.06 – 0.57		2.15 – 2.60	2.49 – 3.02	-0.003 – 0.77		
CV (%)	17.38	21.48			23.33	19.34			25.10	25.71			

Linoleic acid, 18:2n-6 (%)												
Mean ± SD	23.17 ± 2.78	23.32 ± 2.53	0.15 ± 2.74		23.62 ± 3.38	22.64 ± 2.77	-0.98 ± 2.87		24.96 ± 3.48	22.99 ± 2.01	-1.97 ± 2.50		
95% CI	22.10 – 24.24	22.35 – 24.29	-0.90 –1.20	0.68	22.35 – 24.90	21.59 – 23.68	-2.07 – 0.10	0.06	23.65 – 26.28	22.23 – 23.75	-2.92 – -1.03	0.001	0.74
CV (%)	12.01	10.83			14.29	12.23			13.96	8.74			
Dihomo-γ linolenic acid, 20:3n-6 (%)												
Mean ± SD	3.00 ± 0.84	2.76 ± 0.93	-0.24 ±0.91		3.10 ± 1.07	2.67 ± 0.82	-0.42 ± 0.77		2.83 ± 0.87	2.68 ± 0.83	-0.15 ± 0.78		
95% CI	2.67 – 3.32	2.40 – 3.11	-0.59 – 0.11	0.34	2.69 – 3.50	2.36 – 2.98	-0.71 – -0.13	0.007	2.50 – 3.16	2.36 – 2.99	-0.45 – 0.14	0.22	0.91
CV (%)	28.12	33.73			34.64	30.83			30.65	31.02			
Arachidonic acid, 20:4n-6 (%)												
Mean ± SD	10.52 ± 1.99	10.39 ± 2.41	-0.13 ±2.62		11.19 ± 2.32	10.37 ± 2.14	-0.83 ± 1.15		10.81 ± 1.77	10.11 ± 1.83	-0.70 ± 2.09		
95% CI	9.75 – 11.29	9.47 – 11.32	-1.13 – 0.88	0.34	10.32 – 12.07	9.56 – 11.17	-1.26 – -0.39	0.002	10.14 – 11.48	9.42 – 10.80	-1.49 – 0.09	0.06	0.91
CV (%)	18.95	23.20			20.70	20.69			16.40	18.14			
Eicosapentaenoic acid, 20:5n-3 (%)												
Mean ± SD	0.44 ± 0.26	0.50 ± 0.22	0.06 ± 0.25		0.32 ± 0.16	0.49 ± 0.39	0.17 ± 0.41		0.35 ± 0.23	0.44 ± 0.20	0.09 ± 0.28		
95% CI	0.34 – 0.54	0.42 – 0.59	-0.04 – 0.16	0.17	0.26 – 0.38	0.35 – 0.64	0.02 – 0.33	0.07	0.26 – 0.44	0.37 – 0.52	-0.01 – 0.20	0.05	0.43
CV (%)	59.36	44.04			49.31	78.58			65.67	45.38			
Adrenic acid, 22:4n-6 (%)												
Mean ± SD	0.41 ± 0.13	0.36 ± 0.15	-0.06 ±0.18		0.46 ± 0.17	0.37 ± 0.13	-0.09 ± 0.15		0.45 ± 0.18	0.37 ± 0.16	-0.08 ± 0.25		
95% CI	0.36 – 0.46	0.30 – 0.42	-0.12 – 0.01	0.07	0.39 – 0.54	0.32 – 0.42	-0.15 – -0.03	0.004	0.38 – 0.52	0.31 – 0.43	-0.17 – 0.02	0.05	0.63
CV (%)	31.63	41.69			36.95	36.40			40.81	43.72			
Docosapentaenoic acid, 22:5n-3 (%)												
Mean ± SD	0.63 ± 0.23	0.56 ± 0.29	-0.07 ± 0.26		0.57 ± 0.13	0.50 ± 0.18	-0.07 ± 0.17		0.51 ± 0.16	0.57 ± 0.51	0.05 ± 0.50		
95% CI	0.54 – 0.71	0.45 – 0.67	-0.17 –0.03	0.12	0.52 – 0.62	0.43 – 0.57	-0.14 – -0.01	0.06	0.45 – 0.57	0.38 – 0.76	-0.14 – 0.24	0.55	0.80
CV (%)	36.91	52.43			22.30	36.62			31.65	89.43			
Docosahexaenoic acid, 22:6n-3 (%)												
Mean ± SD	3.12 ± 0.86	3.11 ± 0.99	-0.01 ±0.59		2.76 ± 0.86	2.81 ± 0.96	0.06 ± 1.13		2.68 ± 0.92	2.80 ± 0.93	0.12 ± 1.04		
95% CI	2.79 – 3.45	2.72 – 3.49	-0.24 – 0.21	0.60	2.43 – 3.08	2.45 – 3.17	-0.37 – 0.48	0.61	2.33 – 3.03	2.45 – 3.15	-0.27 – 0.52	0.43	0.50
CV (%)	27.59	31.97			31.32	34.12			34.28	33.15			
Ratio Arachidonic/Eicosapentaenoic acid												
Mean ± SD	30.35 ± 18.06	24.18 ± 10.38	-6.17 ±20.09		41.46 ± 17.38	31.88 ± 25.21	-9.58 ± 27.83		45.25±29.89	28.55 ± 15.96	-16.70 ± 32.71		
95% CI	23.41 – 37.29	20.19 – 28.17	-13.89 – 1.55	0.16	34.90 – 48.02	22.37 – 41.39	-20.08 – 0.92	0.03	33.98 –56.53	22.53 – 34.57	-29.04 – -4.36	0.008	0.51
CV (%)	59.50	42.95			41.92	79.07			66.04	55.91			
Ratio Arachidonic/Docosahexaenoic acid												
Mean ± SD	3.57 ± 0.93	3.62 ± 1.23	0.05 ± 0.85		4.40 ± 1.43	4.09 ± 1.61	-0.31 ± 1.90		4.48 ± 1.61	3.97 ± 1.47	-0.50 ± 1.55		
95% CI	3.21 – 3.92	3.15 – 4.09	-0.27 – 0.38	0.95	3.85 – 4.94	3.48 – 4.69	-1.03 – 0.41	0.34	3.87 – 5.09	3.42 – 4.53	-1.09 – 0.08	0.10	0.77
CV (%)	25.99	33.91			32.64	39.30			36.06	37.09			
Ratio n-6/n-3 polyunsaturated fatty acids												
Mean ± SD	9.57 ± 2.84	9.70± 3.41	0.13±1.84		11.29±3.42	10.51 ± 3.44	-0.78±4.59		11.97 ± 3.61	10.55 ±3.42	-1.41±3.61		
95% CI	8.42– 10.71	8.32 – 11.07	-0.61–0.87	0.60	9.94 – 12.64	9.15– 11.87	-2.59–1.04	0.40	10.54 – 13.39	9.20 –11.91	-2.84–0.01	0.06	0.46
CV (%)	29.28	34.44			30.28	32.73			30.16	32.42			
Total saturated fatty acids (%)												
Mean ± SD	47.63 ± 2.08	48.29 ± 2.31	0.66 ± 3.59		47.08 ± 2.90	49.17 ± 1.50	2.09 ± 3.56		46.53 ± 3.01	48.96 ± 1.75	2.44 ± 3.74		
95% CI	46.83 – 48.43	47.40 – 49.18	-0.72 – 2.04	0.32	45.99 – 48.18	48.61 – 49.74	0.75 – 3.44	0.005	45.39 –47.66	48.30 – 49.62	1.02 – 3.85	0.002	0.65
CV (%)	4.37	4.79			6.17	3.05			6.47	3.57			
Total monounsaturated fatty acids (%)												

Mean ± SD	11.08 ± 1.16	10.70 ± 1.74	-0.38 ±2.12		10.98 ± 1.34	10.98 ± 1.46	0.001 ± 1.89		10.87 ± 1.45	11.05 ± 1.66	0.18 ± 1.84		
95% CI	10.63 – 11.52	10.03 – 11.37	-1.19 –0.43	0.29	10.47 –11.49	10.43 – 11.53	-0.71 –0.71	0.96	10.33 –11.42	10.43 – 11.68	-0.51 – 0.87	0.85	0.69
CV (%)	10.51	16.26			12.21	13.28			13.33	15.02			
Total polyunsaturated fatty acids (%)													
Mean ± SD	41.29 ± 2.17	40.99 ± 2.25	-0.30 ±3.23		42.02 ± 3.04	39.85 ± 1.25	-2.17 ±3.06		42.60 ± 3.11	39.96 ± 1.89	-2.64 ± 3.64		
95% CI	40.46 – 42.12	40.13 – 41.86	-1.54 – 0.95	0.57	40.87 – 43.16	39.38 – 40.32	-3.32 – -1.01	0.001	41.43– 43.77	39.25 – 40.68	-4.01 – -1.26	0.001	0.36
CV (%)	5.25	5.49			7.23	3.13			7.29	4.74			
Total n-3 polyunsaturated fatty acids (%)													
Mean ± SD	4.19 ± 1.16	4.17 ± 1.21	-0.02 ± 0.74		3.65 ± 0.95	3.80 ± 1.28	0.16 ± 1.35		3.54 ± 1.06	3.81 ± 1.32	0.27 ± 1.37		
95% CI	3.74 – 4.63	3.70 – 4.63	-0.30 – 0.26	0.87	3.29 – 4.00	3.32 – 4.28	-0.35 – 0.67	0.94	3.14 – 3.94	3.32 – 4.31	-0.25 – 0.79	0.25	0.29
CV (%)	27.69	29.03			26.16	33.57			29.89	34.55			
Total n-6 polyunsaturated fatty acids (%)													
Mean ± SD	37.10 ± 2.25	36.83 ± 2.19	-0.28 ±3.20		38.37 ± 2.63	36.05 ± 1.29	-2.33 ±2.93		39.06 ± 3.10	36.15 ± 1.89	-2.91 ± 3.37		
95% CI	36.24 – 37.97	35.98 – 37.67	-1.51 –0.96	0.68	37.38 – 39.37	35.56 – 36.53	-3.43 – -1.22	0.0005	37.89– 40.23	35.44 – 36.86	-4.18 – -1.63	0.0005	0.62
CV (%)	6.07	5.96			6.87	3.57			7.94	5.23			
Delta-5 desaturase activity													
Mean ± SD	3.88 ± 1.58	4.26 ± 2.00	0.38 ± 1.84		4.05 ± 1.60	4.14 ± 1.26	0.10 ± 1.21		4.20 ± 1.53	4.19 ± 1.66	-0.01 ± 1.37		
95% CI	3.27 – 4.48	3.49 – 5.03	-0.32 –1.09	0.71	3.44 – 4.65	3.67 – 4.62	-0.36 – 0.55	0.37	3.62 – 4.78	3.56 – 4.82	-0.52 – 0.51	0.90	0.91
CV (%)	40.70	46.92			39.51	30.50			36.39	39.70			
Delta-6 desaturase activity													
Mean ± SD	0.13 ± 0.05	0.12 ± 0.04	-0.01 ±0.04		0.14 ± 0.06	0.12 ± 0.05	-0.01 ± 0.04		0.12 ± 0.05	0.12 ± 0.04	0.001 ± 0.04		
95% CI	0.12 – 0.15	0.10 – 0.14	-0.03 – 0.003	0.39	0.11 – 0.16	0.10 – 0.14	-0.03 – 0.002	0.09	0.10 – 0.14	0.10 – 0.13	-0.01 – 0.02	0.98	0.95
CV (%)	34.48	37.27			42.51	39.40			40.01	34.47			

Values are mean ± standard deviation (SD), 95% confidence interval (CI) and coefficient of variation (CV). Change (Δ) is post-baseline. Intragroup (IG) differences were estimated by the non-parametric Wilcoxon test and between groups (BG) differences were estimated by the non-parametric Kruskal-Wallis test. All the *p*-values are indicated.

Table S7. Habitual daily intake of total energy, main nutrients and fatty acids of the sample population included in the study and distributed into the placebo (PLB) and chokeberry (AMJ and AMJ_d) intervention groups.

	PLB (n=29)	AMJ (n=27)	AMJ_d (n=28)	*p*-value (between groups)
Energy (Kcal)				
Mean ± SD	2075.09 ± 597.27	2055.30 ± 499.27	2015.90 ± 555.04	
Median	2087.48	1901.62	1931.40	0.94
95% CI	1853.86 – 2296.32	1866.98 –2243.63	1806.53 – 2225.26	
CV (%)	28.78	24.29	27.53	
Main macronutrients				
Fat (g)				
Mean ± SD	95.26 ± 33.01	89.25 ± 28.83	94.15 ± 33.84	
Median	97.30	81.21	86.57	0.81
95% CI	83.04 – 107.49	78.37 – 100.12	81.38 – 106.92	
CV (%)	34.65	32.30	35.95	
Protein (g)				
Mean ± SD	82.05 ± 25.24	80.81 ± 21.48	79.82 ± 24.81	
Median	86.65	76.96	75.31	0.86
95% CI	72.70 – 91.40	72.71 – 88.91	70.46 – 89.18	
CV (%)	30.77	26.58	31.08	
Carbohydrates (g)				
Mean ± SD	222.38 ± 77.41	226.75 ± 68.25	212.31 ± 61.89	
Median	219.12	202.82	204.76	0.82
95% CI	193.71 – 251.05	201.01– 252.49	188.97– 235.66	
CV (%)	34.81	30.10	29.15	
Main types of fatty acids				
Saturated fatty acids (g)				
Mean ± SD	28.54 ± 11.17	30.22 ± 12.08	31.50 ± 13.73	
Median	29.45	27.52	28.78	0.82
95% CI	24.40 – 32.68	25.66 – 34.77	26.32 – 36.68	
CV (%)	39.14	39.98	43.58	
Monounsaturated fatty acids (g)				
Mean ± SD	30.13 ± 11.66	28.27 ± 10.44	29.28 ± 12.93	
Median	28.03	25.37	25.71	0.84
95% CI	25.81 – 34.45	24.33 – 32.21	24.40 – 34.15	
CV (%)	38.70	36.92	44.15	
Polyunsaturated fatty acids (g)				
Mean ± SD	26.86 ± 13.67	22.29 ± 8.88	23.86 ± 8.63	
Median	24.23	20.05	23.83	0.60
95% CI	21.80 – 31.93	18.94 – 25.64	20.60 – 27.12	
CV (%)	50.89	39.82	36.19	

Values are mean ± standard deviation (SD), median, 95% confidence interval (CI) and coefficient of variation (CV). Between groups differences were estimated by the non-parametric test of Kruskal-Wallis. All the *p*-values are indicated.
